# Faecal bacterial microbiota in patients with cirrhosis and the effect of lactulose administration

**DOI:** 10.1186/s12876-017-0683-9

**Published:** 2017-11-28

**Authors:** Aditya Narayan Sarangi, Amit Goel, Ankur Singh, Avani Sasi, Rakesh Aggarwal

**Affiliations:** 1 Department of Gastroenterology, Sanjay Gandhi Postgraduate Institute of Medical Sciences, Lucknow, 226014 India; 2 Biomedical Informatics Center, Sanjay Gandhi Postgraduate Institute of Medical Sciences, Lucknow, India

**Keywords:** Cirrhosis, Dysbiosis, Gut microbiota, Hepatic encephalopathy, Lactulose

## Abstract

**Background:**

Gut microbiota may be altered in patients with cirrhosis, and may further change after administration of lactulose. We studied the composition of gut microbiota in patients with cirrhosis and assessed the effect on it of lactulose administration.

**Methods:**

Stool specimens were collected from 35 patients with cirrhosis (male 26; median [range] age: 42 [29–65] years) and 18 healthy controls (male 14; 44.5 [24–67] years); 21 patients provided another specimen after lactulose administration for 55 [42–77] days. For each, a DNA library of V3 region of bacterial 16S ribosomal RNA was subjected to paired-end Illumina sequencing. Inter-specimen relationship was studied using principal co-ordinate analysis. Abundances of various bacterial taxa, and indices of alpha and beta diversity were compared, between patients and controls, and between specimens collected before and after lactulose.

**Results:**

Gut microbiota from cirrhosis patients and controls showed differential clustering, and microbiota from patients with cirrhosis had less marked alpha diversity. Abundances of dominant phyla (Bacteroidetes, Firmicutes and Proteobacteria) were similar. However, patients with cirrhosis had lower abundances of five phyla, namely Tenericutes, Cyanobacteria, Spirochaetes, Elusimicrobia and Lentisphaerae, and differences in abundances of several families and genera than in controls. Lactulose administration did not lead to any change in alpha and beta diversities, species richness and abundances of various bacterial taxa in gut microbiota.

**Conclusions:**

Gut microbiota in cirrhosis differ from healthy persons and do not change following lactulose administration. The latter suggests that the effect of lactulose on hepatic encephalopathy may not be related to alteration in gut microbiota.

**Electronic supplementary material:**

The online version of this article (10.1186/s12876-017-0683-9) contains supplementary material, which is available to authorized users.

## Background

Cirrhosis is characterized by fibrosis leading to altered liver architecture, resulting in marked reduction in its function and in portal hypertension. Patients with cirrhosis are prone to serious complications, such as variceal gastrointestinal bleeding, hepatic encephalopathy (HE), ascites, spontaneous bacterial peritonitis, hepatorenal syndrome, hepatocellular carcinoma, and an increased mortality.

Normal human gut is inhabited by several microorganisms, in particular bacteria. The number of bacterial cells in the intestinal lumen of an individual is of the order of that of human cells in the body. These bacteria perform several important physiologic functions, such as digestion of complex carbohydrates leading to energy salvage, synthesis of essential substances such as vitamin K, and modulation of mucosal and systemic immune responses [1]. With its strategic placement between the bowel and the systemic circulation, liver acts as a filter and removes any bacteria and their harmful products that may enter the blood from the gut [2]; the loss of this function in patients with cirrhosis may play a role in the occurrence of complications such as spontaneous bacterial peritonitis, sepsis and hepatorenal syndrome [3].

Modulation of gut microbiota using antimicrobial agents [4] and probiotic preparations [5] has been shown to improve HE; this suggests that intestinal bacteria play a role in the causation of HE. Lactulose, a non-absorbable disaccharide, is another drug used for the prevention and treatment of HE. It cannot be digested by human intestinal enzymes, and reaches the colon unchanged where it is degraded by bacteria. It is believed that the resultant acidic environment changes the composition of the gut microbiota [6, 7], with a reduction in the bacteria that produce ammonia and an increase in those that trap and use ammonia for their metabolism [7], ameliorating HE. In fact, in studies using stool culture, lactulose administration has been shown to alter the abundance of certain gut bacteria [8, 9]. However, culture-based techniques have several limitations. In particular, a majority of bacterial species inhabiting the gut can either not be cultured or not be reliably distinguished from other related bacteria. Further, these techniques are only qualitative or, at best, semi-quantitative. Thus, there is a need to study the effect of lactulose on the composition of gut microbiota using better techniques.

In recent years, high-throughput sequencing of gene for bacterial 16S ribosomal RNA has emerged as a useful method for studying the composition of complex bacterial mixtures [10]. These techniques exploit the differences in this gene between various bacteria, such that their sequences accurately identify various bacterial groups, often up to the species level. Further, these techniques can sequence several DNA molecules in parallel, providing data on relative abundance of different bacteria in a mixture. In the current study, we applied high-throughput sequencing to determine whether gut microbiota in patients with cirrhosis differed from those of healthy controls, and whether lactulose treatment leads to a change in the composition of gut microbiota in patients with cirrhosis.

## Methods

### Subjects

Patients with cirrhosis, irrespective of the cause or severity of liver dysfunction, measured using Child-Turcotte-Pugh (CTP) class, and with no co-existing disease, were enrolled from the outpatient clinic of our institution between October 2013 and April 2014. Diagnosis of cirrhosis was based on a combination of typical clinical, biochemical, endoscopic and radiological findings. Patients who had taken drugs that can influence the gut microbiota, such as gastric acid suppressants, antimicrobial agents, probiotics, non-absorbable disaccharides (such as lactulose) or those that alter gastrointestinal motility, or complementary or alternative medicines, in the previous 6 weeks, were excluded. Since we also aimed to study the effect of lactulose on gut microbiota, we particularly included those patients who were likely to be prescribed lactulose, as a prophylaxis against HE in view of CTP class B or C disease.

For each patient, one stool specimen was collected at enrollment. For patients who received lactulose, a second specimen was collected after 6 weeks of lactulose administration (in a dose of 30–60 ml/day, adjusted to obtain 2–3 semisolid stools daily); any patient who had worsening of clinical condition, or received another medication or required hospitalization was excluded.

In addition, a group of healthy adult volunteers, with similar age and gender distribution, were recruited as controls from among family members of other patients presenting with minor illnesses (so that they were similar to the patients in terms of socioeconomic status, diet, lifestyle, habits, etc). The prospective control subjects underwent recording of clinical history and a physical examination by a physician, and those with any current symptom/illness or any significant previous illness, and those receiving any drug likely to affect gut microbiota were excluded. Each control subject provided one stool specimen.

The subjects, both patients and controls, collected a stool specimen in a wide-mouthed container at site (in the hospital) and placed it immediately in a box containing cool-packs which had been frozen at −80 °C. The study was approved by the Ethics Committee of Sanjay Gandhi Postgraduate Institute of Medical Sciences, Lucknow and each participant provided informed consent.

### Sequencing of gut microbiota

DNA was extracted from approximately 0.5 g of stool using standard phenol-chloroform method, and V3 hypervariable region of 16S rRNA gene was amplified [11]. These primers, in addition to the V3-specific priming sequences, contained sequences complementary to Illumina forward, reverse and multiplex sequencing primers. Different reverse primers, each with a unique six-nucleotide index, were used for different stool specimens to enable multiplexing (Table 1).

**Table 1:** Custom primers used for generation of Illumina DNA libraries

Primer name	Primer nucleotide sequence
V3F	5′-aatgatacggcgaccaccgagatctacactctttccctacacgacgctcttccgatct **NNNN**CCTACGGGAGGCAGCAG-3′
V3R	5′-caagcagaagacggcatacgagat**XXXXX** gtgactggagttcagacgtgtgctcttccgatctATTACCGCGGCTGCTGG-3′

Lower case letters represent adapter sequences necessary for binding to Illumina flow cell, underlined lowercase letters represent binding site for Illumina sequencing primers, and upper case letters represent V3 region primers (341F on the forward and 518R on the reverse primer). NNNN represents degenerate bases for adding sequence diversity necessary for proper cluster identification by the sequencer, and XXXXXX represents the 6-nucleotide index region for multiplexing

Illumina sequencing libraries were prepared using a one-step polymerase chain reaction in a 50-μl reaction mixture that contained 200 ng of input DNA, 6.25 pmol each of forward and reverse primers and KAPA Hi-Fi PCR master mix (Kapa Biosystems, Boston, MA, USA). The PCR conditions were: an initial denaturation at 95 °C for 5 min, followed by 20 cycles of 95 °C, 65 °C, and 72 °C for 1 min each, and a final extension at 72 °C for 5 min. The amplification products were purified using 2% agarose (in tris-borate-ethylenediaminetetraacetic acid) gel electrophoresis, followed by recovery of amplicons of desired length (GenElute Gel extraction kit; Sigma-Aldrich). Purified libraries were checked for size distribution, quantitated (Agilent Bioanalyser DNA1000) and normalized to 10 nM. The normalized libraries were pooled in sets of 8–12 specimens each and sequenced in one lane of an Illumina HiScan SQ sequencing flow cell using standard 2 X 101-cycle paired-end multiplex sequencing format. Library pool was spiked with 30% Illumina PhiX control library to enhance sequence diversity for efficient base calling. Data were then demultiplexed using Illumina CASAVA software.

### Processing of sequence data

The raw reads in opposite directions were merged using PANDAseq software [12], and primer sequences were trimmed out. Sequences shorter than 100 nucleotides, with any ambiguous nucleotide, or with an overlap of fewer than 20 nucleotides in paired reads were purged. The merged reads were subjected to quality control using NGSQC Toolkit [13], to exclude those with average Phred quality score below 30. The selected high-quality reads were processed using Quantitative Insights into Microbial Ecology (QIIME V1.8) software package [14]. Any chimeric sequences, identified using Usearch61, were purged. The remaining reads were assigned to operational taxonomic units (OTUs) using UCLUST-based sub-sampled open-reference OTU picking protocol [15]. A representative sequence for each OTU was aligned with the Greengenes core set alignment using the PyNAST tool [16]; any sequences that failed to align were purged. Based on alignment of the representative OTU sequences, a phylogenetic tree was constructed using the FastTree tool [17]. Taxonomy was assigned to each OTU using the QIIME’s UCLUST Consensus Taxonomy Assigner against the Greengenes v13.8 reference OTUs, using the software’s default parameters. Thereafter, to reduce noise, OTUs that were observed in fewer than 10% of stool specimens (*n* = 5) or accounted for fewer than 0.002% of reads in all the specimens taken together were purged out. Sample-wise observation count of each OTU was tabulated as an OTU table in ‘biom’ format. The filtered OTU table was then used for determination of bacterial composition of each sample.

### Alpha diversity analysis

OTU table was rarefied, using PhyloSeq [18], to equalize the sampling depth of all the specimens to the one with the fewest reads. For each specimen, alpha diversity was estimated using Chao1 and Abundance-based Coverage Estimator indices, which measure the species richness, and using Shannon and Simpson indices, which measure the richness and distribution of taxa [19]. These indices were compared between groups using the compare_alpha_diversity.py script of QIIME 1.8, with Bonferroni correction.

### Beta diversity analysis

The filtered data were assembled into a table where each row represented an OTU and each column represented a faecal specimen. The cells contained observation counts for a particular OTU in a particular specimen, normalized using a log-frequency transformation, as follows:

$$ \mathrm{Normalized}\  \mathrm{value}=\kern0.5em {\mathrm{Log}}_{10}\left(\frac{OC}{n}\times \frac{\sum X}{N}+1\right) $$

Where ‘OC’ represents the actual observed count of a particular OTU in a specimen, ‘n’ is the sum of observed counts for all OTUs in the particular specimen, Σx is the sum of ‘n’ across all specimens in the table and N is the total number of specimens in the table.

Beta diversity was assessed using principal co-ordinate analysis (PCoA) on weighted UniFrac distance matrices generated from the normalized OTU tables.

### Comparison of gut microbiota composition between groups

Abundances of various bacterial taxa at different taxonomic levels in patients and controls were compared using Mann-Whitney U test, and those before and after lactulose were compared using paired *t* test. In either case, Benjamini-Hochberg false discovery rate correction was used to account for multiple comparisons, using *p* < 0.05 as the cut-off.

### Cirrhosis-dysbiosis ratio

This ratio, a previously-described quantitative index of microbiota alterations accompanying cirrhosis progression, was computed as the natural log (ln) of the ratio of aggregated abundance of autochthonous (Lachnospiraceae, Ruminococcaceae and Veillonellaceae) and non-autochthonous (Enterobacteriaceae and Bacteroidaceae) taxa [20]. Data from patients and controls were compared using Mann-Whitney U test, and those before and after lactulose using Wilcoxon’s signed-rank test.

### PICRUSt analysis

Putative metabolic functions of the microbial communities in each specimen were predicted using PICRUSt (Phylogenetic Investigation of Communities by Reconstruction of Unobserved States) [21]. This tool compares the identified 16S rRNA gene sequences with the annotated genome sequences of known species, thereby estimating the possible gene content of a particular microbial community. In brief, OTUs were picked using the closed-reference OTU picking approach at ≥97% identity against the Greengenes database (version 13.5) using QIIME 1.8. The OTU table was then normalized for 16S rRNA gene copy numbers and the corresponding metagenomes were predicted. In addition, the proportions of bacteria that would be expected to contain the glutamine gene, and alpha, beta and gamma subunits of the urease gene were also estimated. Putative metabolic functions of the microbial communities in each specimen were compared between patients and controls using Mann-Whitney U test, and those before and after lactulose were compared using Wilcoxon’s signed-rank test.

### Statistical analysis

Clinical and laboratory variables were compared between groups using chi-squared test for categorical data, and unpaired t-test or Mann-Whitney U test for numerical data. *P* values <0.05 were considered significant.

## Results

Baseline stool specimens were collected from 35 patients with cirrhosis (median [range] age: 42 [29–65] years; 26 male; body mass index: 22.8 [17.3–32.3] Kg/m^2^) and 18 controls (44.5 [24–67] y; male 14; 23.3 [20.0–25.0] Kg/m^2^); the clinical and laboratory findings for patients are summarized in Table 2. Thirty of them received regular lactulose administration as a part of standard of care, and 21 of these 30 patients provided repeat stool specimens after lactulose administration for a median of 55 (42–77) days. The baseline characteristics of these 21 patients were similar to those of 14 patients for whom repeat specimens were not available, because they did not receive lactulose, were lost to follow-up, received a proton-pump inhibitor or an antibiotic during the intervening period, or had a clinical worsening or required hospitalization (Additional file 1: Table S1).

**Table 2:** Laboratory parameters and disease severity indices in patients with cirrhosis (*n* = 35)

Parameter	Value	
Hemoglobin (g/dL) (reference: >13.0)	11.0	(6.6–14.7)
Leucocyte count (×1000/μL) (reference: 4.0–7.0)	4.5	(1.9–9.2)
Platelet count (×1000/μL) (reference: 150–400)	75.0	(22–184)
Serum bilirubin (mg/dL) (reference: <1.2)	2.0	(0.5–8.0)
Serum aspartate transaminase (IU/L) (reference: <40)	68.0	(26–273)
Serum alanine transaminase (IU/L) (reference: <40)	40.0	(17–162)
Serum alkaline phosphatase (IU/L) (reference: <150)	141.0	(36–455)
Serum albumin (g/dL) (reference: 3.5–5.0)	3.4	(2.1–4.5)
Serum creatinine (mg/dL) (reference: <1.2)	0.9	(0.6–1.8)
Prothrombin time (International normalized ratio)	1.3	(0.9–2.8)
Child-Turcotte-Pugh score	7.0	(5–11)
Model for end-stage liver disease (MELD) score	13.0	(6–25)
Cause of liver disease		
Hepatitis C	9	
Hepatitis B	7	
Alcohol	7	
Autoimmune	1	
Cryptogenic	11	
Child-Pugh class		
A	10	(29%)
B	21	(60%)
C	4	(11%)
Clinical history		
Ascites	19	(54%)
Spontaneous bacterial peritonitis	6	(32%)
Hepatic encephalopathy	4	(11%)
Variceal bleed	8	(23%)

Data are shown as median (range) or as number (%)

The 74 stool specimens studied (18 controls; 35 pre-lactulose and 21 post-lactulose) yielded a total of 40,023,099 high-quality reads (median [range] = 474,267 [131,802–1,267,206]), their rarefaction curves are shown in Additional file 2: Fig. S1.

### Gut microbiota in healthy controls versus patients with cirrhosis

The patients and controls were comparable in age, gender distribution, and BMI. All the participants consumed predominantly vegetarian diet.

**Fig. 1:**
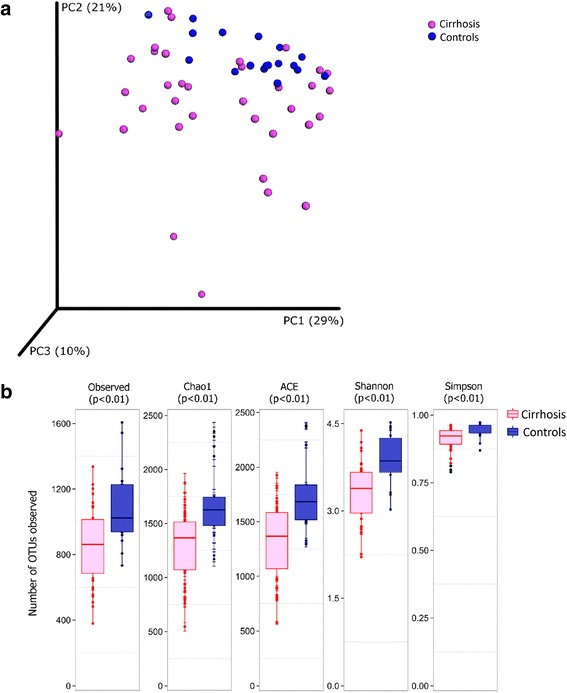
Alpha and beta diversity analysis for gut microbiota from patients with cirrhosis versus healthy controls. *Legend*: **a**: Principal co-ordinate analysis (beta diversity) of weighted UniFrac distances for specimens from patients with cirrhosis (red) and healthy controls (blue). **b**: Comparison of measures of alpha diversity between specimens from cirrhosis patients (red) and controls (blue). The *p* values were obtained using nonparametric t-test with Bonferroni correction. OTU: operational taxonomic unit

In PCoA, faecal specimens from patients showed a wider spread than those from control subjects, indicating a more marked intra-group diversity of microbiota among patients. Further, the specimens from controls showed differential clustering than those from patients with cirrhosis (Fig. 1a). The patient specimens also showed lower alpha diversity and species richness than those from controls **(**Fig. 1b, Additional file 3: Table S2**)**.

**Fig. 2:**
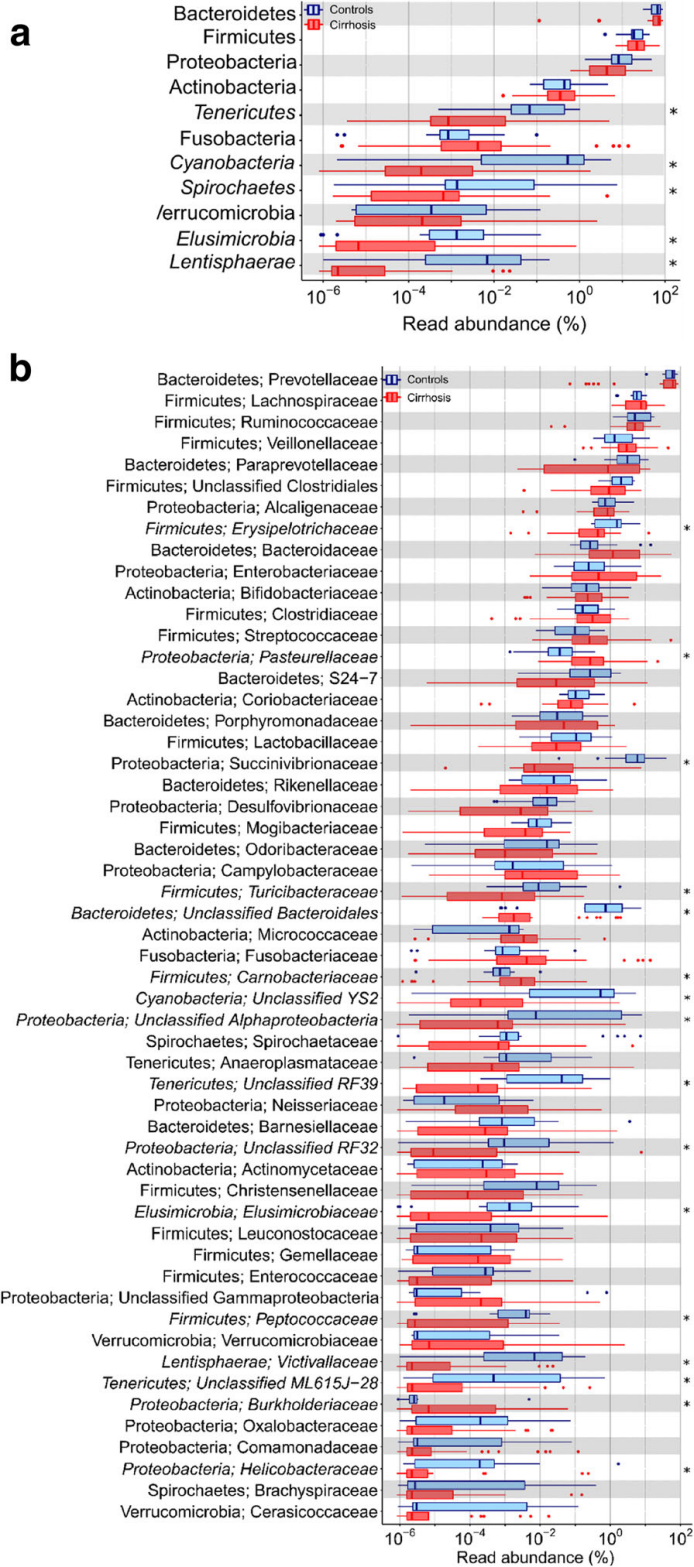
Abundances of various bacterial phyla and families among gut microbiota in cirrhosis and controls. *Legend:* **a**: Abundances of various phyla in patients with cirrhosis (red) and healthy controls (blue). **b**: Abundances of selected families in patients with cirrhosis (red) and healthy controls (blue). All data are shown using box-plots (25th to 75th percentiles) and percent values on a log_10_ scale. Any dots to the left or right of the boxes indicate outliers. Asterix marks indicate values with significant difference between patients and controls (*p* < 0.05; Mann-Whitney U test with Benjamini-Hochberg correction

Median [range] number of high-quality reads for 18 controls and 35 baseline cirrhosis specimens were 407,482 (316,749–1,139,360; total reads 9,388,696) and 504,182 (131,802–1,267,206; total 19,466,867), respectively. These reads belonged to 16,357 non-singleton OTUs. Of these, 855 OTUs belonging to 11 phyla were identified in at least five specimens each and formed >0.002% of the total reads, and were analyzed further. Abundances of the dominant phyla, namely Bacteroidetes (71.91% [0.11–90.01] vs 66.82% [30.35–88.99]), Firmicutes (21.95% [6.95–74.56] vs. 18.65% [3.95–43.47]) and Proteobacteria (4.37% [0.61–50.64] vs. 8.20% [1.34–48.35]) were comparable in patients and controls. However, five phyla, namely Tenericutes [0% vs 0.07%], Cyanobacteria [0% vs 0.53%], Spirochaetes [0.00065% vs 0.0014%], Elusimicrobia [0% vs 0.0013%] and Lentisphaerae [0% vs 0.007%] were less abundant in patients than in controls (Benjamini-Hochberg corrected *p*-values <0.05 for each) (Fig. 2a, Additional File 4: Table S3**).** Similarly, some specific classes, orders, families and genera had significantly different abundances in patients and controls (Fig. 2b; Additional file 5: Figure S2a, S2b and S2c; Additional file 4: Table S3).

**Fig. 3:**
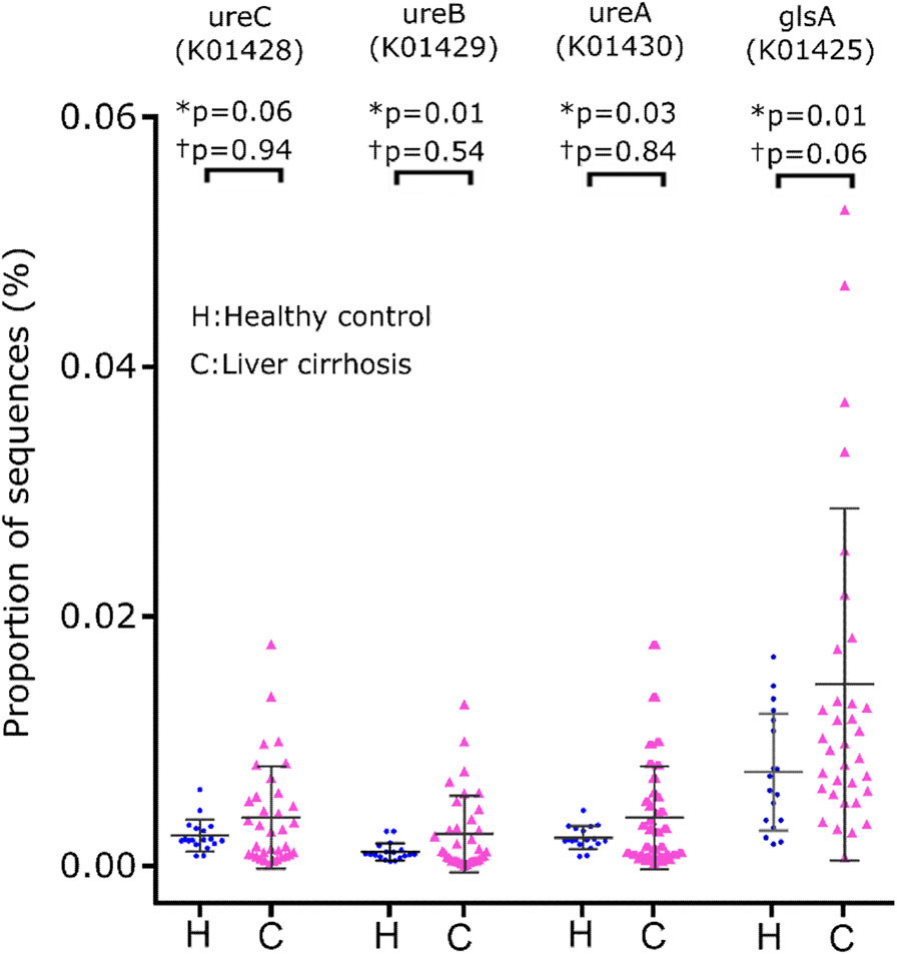
Abundance of bacteria predicted to contain glutaminase gene and various subunits of the urease gene. *Legend:* Scatter plots showing comparison of abundance in gut microbiota of healthy controls (blue) and cirrhosis (red) of the bacteria predicted to contain glutaminase gene glsA, K01425) and urease gene subunits alpha (ureC, K01428), beta (ureB, K01429) and gamma (ureA, K01428) genes. Data are presented as mean ± SD. *P* values were calculated using both parametric (unpaired t-test, marked as *) and non-parametric (Mann-Whitney test, marked as †) methods, followed by Welch’s correction for multiple testing

Cirrhosis-dysbiosis ratio was significantly lower in patients with cirrhosis than in controls (1.55 ± 1.86 versus 2.71 ± 1.48; *p* = 0.019, Mann-Whitney U test). The abundances of bacteria that are predicted to contain the glutaminase gene or genes for alpha, beta or gamma subunits of urease were more widely distributed and somewhat higher in patients than in controls (Fig. 3), but the inter-group comparisons did not show a significant difference (Fig. 3).

### Gut microbiota in patients with liver cirrhosis before and after lactulose use

**Fig. 4:**
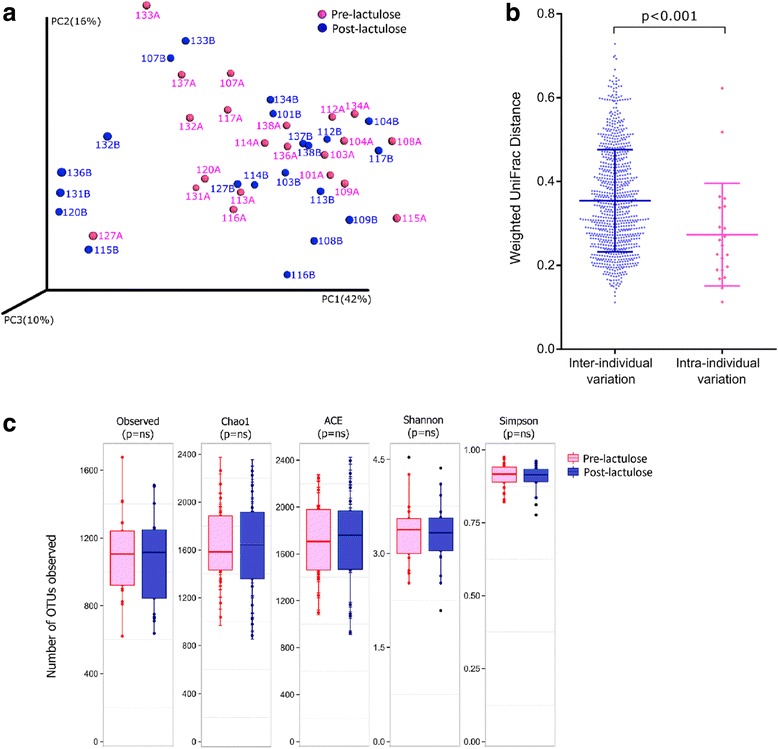
Alpha and beta diversity of gut microbiota in patients with cirrhosis, before and after lactulose. *Legend:* **a**: Principal co-ordinate analysis of weighted UniFrac distances between faecal specimens collected from patients with cirrhosis before (red) and after (blue) lactulose administration. **b**: Comparison of weighted UniFrac distances between individual pre-lactulose specimens from different patients (left, in blue) and between paired (pre- and post-lactulose) specimens from each patient (right, in red); the latter distances were significantly less (*p* < 0.001) than the former. **c**: Comparison of measures of alpha diversity between specimens from patients with cirrhosis collected before (red) and after (blue) lactulose administration. The *p* values shown were obtained using Mann-Whitney U test with Bonferroni correction

Paired stool specimens, before and after lactulose, were available for 21 patients (median age: 45 [29–64] years; male 13; Child-Pugh class A: 7, B: 13, C: 1). Median [range] number of high-quality reads in their specimens before and after lactulose were similar (465,630 [131,802–1,231,311] versus 446,762 [159,321–1,022,517]). On PCoA, the distributions of data points for specimens collected before or after lactulose overlapped. Data points for specimens collected before and after lactulose for individual subjects were located close to each other (Fig. 4a); the weighted UniFrac distances between paired specimens were smaller than those between individual pre-lactulose specimens (Fig. 4b). None of the indices of alpha diversity showed a significant change following lactulose (Fig. 4c).

**Fig. 5:**
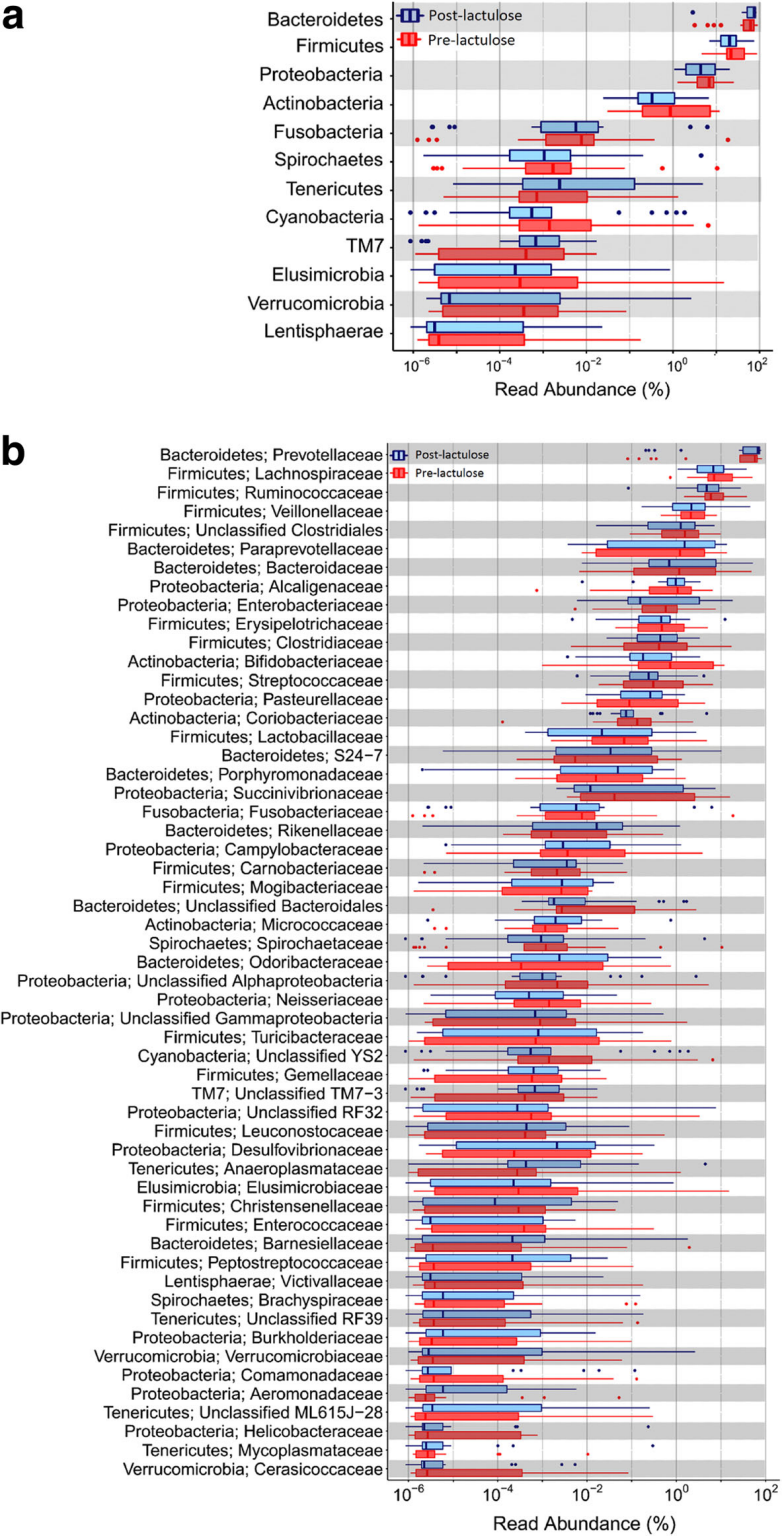
Abundances of various bacterial groups in gut microbiota before and after lactulose administration. *Legend:* Abundances of various bacterial groups among gut microbiota from in patients with liver cirrhosis before (red) and after lactulose (blue) at phylum (**a**) and family (**b**) levels. Data are shown using box-plots (25th to 75th percentiles) and percent values on a log_10_ scale. Any dots to the left or right of the boxes indicate outliers. No bacterial groups showed a significant difference (p value cut-off = 0.05; paired t test with Benjamini-Hochberg correction)

High-quality reads from the 42 paired specimens belonged to 15,209 non-singleton OTUs; of these, 741 OTUs which were identified in at least 10% specimens (four specimens) each and formed >0.002% of the total reads were analysed further. The abundances of four major phyla were similar in the specimens collected before and after lactulose, namely Bacteroidetes (75.55% [2.86–84.49] versus 61.79% [3.16–90.99]), Firmicutes (20.27% [6.93–74.93] versus 21.72% [4.62–86.93]), Proteobacteria (4.37% [1.08–20.37] versus 6.82% [1.27–25.45]) and Actinobacteria (0.33% [0.02–6.74] versus 0.87% [0.03–11.92]). Further, no difference was found in abundances of various phyla, classes, orders, families or genera between the specimens collected before and after lactulose (Fig. 5a and b; Additional file 6: Figure S3a, S3b and S3c).

**Fig. 6:**
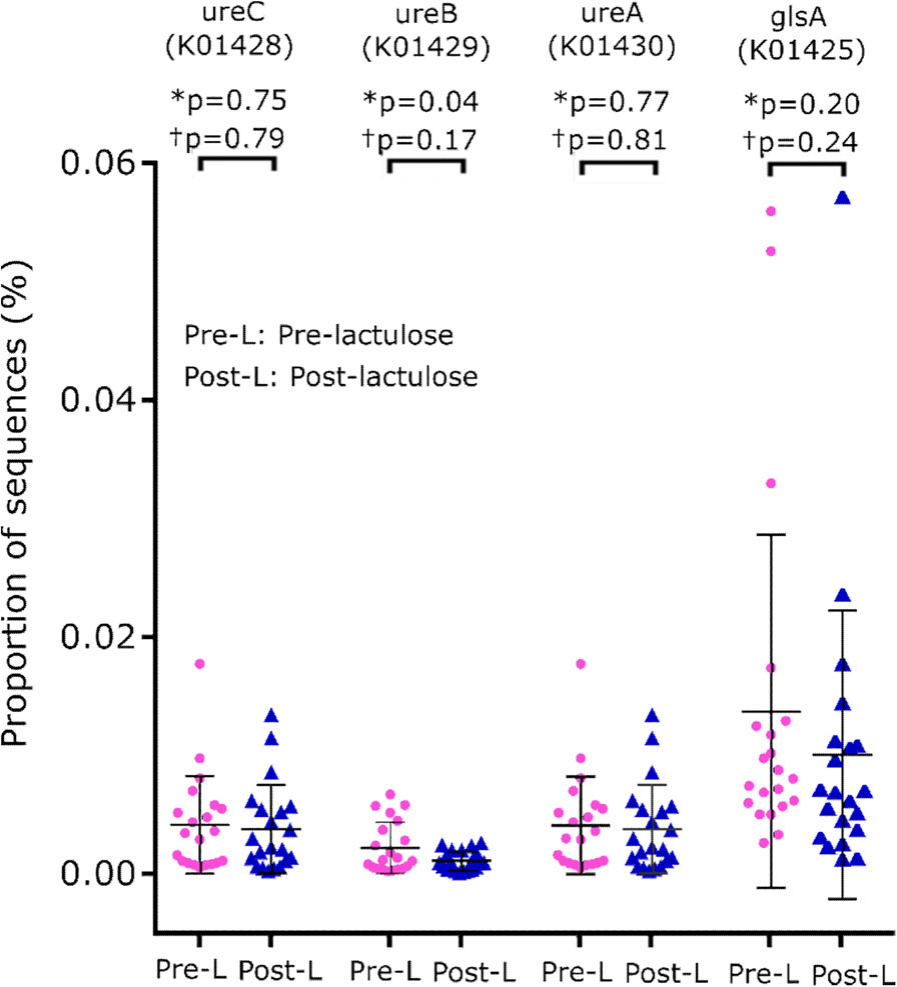
Abundance of bacteria with glutaminase gene and subunits of urease gene before and after lactulose. *Legend:* Comparison of abundances of the bacteria predicted to contain glutaminase gene (glsA, K01425) and urease gene subunits alpha (ureC, K01428), beta (ureB, K01429) and gamma (ureA, K01428) in patients with liver cirrhosis before (Pre-; red) and after (Post-; blue) lactulose administration. Data is presented as mean ± SD. P values were calculated using both parametric (paired t-test, marked as*) and non-parametric (Wilcoxon’s signed rank test, marked as †), followed by Welch correction

Cirrhosis-dysbiosis ratios of faecal specimens before and after lactulose were similar (1.75 ± 1.98 and 2.01 ± 1.70, respectively; *p* = 0.337). The abundances of bacteria predicted to contain genes for glutaminase, or genes for alpha, beta or gamma subunits of urease gene also did not change after lactulose (Fig. 6 **)**.

## Discussion

Using a culture-independent, next-generation sequencing technique, we found that composition of intestinal microbiota in patients with cirrhosis was significantly different from that in healthy persons, as evidenced by differential clustering of patients and healthy persons on PCoA. The patients with cirrhosis had relatively lower abundances of bacteria belonging to phyla Tenericutes, Cyanobacteria, Spirochaetes, Elusimicrobia and Lentisphaerae, and to some specific families and genera. In addition, their gut microbiota had less bacterial diversity and species richness. Further, gut microbiota in patients with cirrhosis before and after administration of lactulose for 6 weeks, showed no difference in composition or diversity.

Healthy human intestine, contains several bacterial species, with a fair degree of inter-individual diversity [21]. For instance, in a study of 124 European individuals, though their faecal specimens taken together harboured between 1000 and 1150 bacterial species, each individual specimen was found to contain only about 160 bacterial species. Several species were shared across individuals, with nearly 75 being common to more than half the subjects; however, the other species were highly variable between individuals. In most people, nearly 90% of the gut bacteria belong to two phyla – Bacteroidetes and Firmicutes, whereas the remaining belong mostly to phyla Proteobacteria, Actinobacteria, Verrucomicrobia and Fusobacteria [22]. The mixture of bacteria and other organisms (such as archaea and fungi) present in an individual’s gut – collectively referred to as the ‘gut microbiota’ – behaves as a metabolic organ and plays an intimate role in regulation and maintenance of normal physiology, metabolism and immune functions. In recent years, changes in gut microbiota – the so-called gut ‘dysbiosis’ – has been implicated in the pathogenesis of several diseases, such as hepatic and gastrointestinal diseases [3], obesity [23], diabetes mellitus [24] and hypertension [25].

We found a difference in the profile of gut microbiota in patients with cirrhosis and healthy persons, as shown by differential clustering on PCoA. Further, the patients with cirrhosis had less diverse gut microbiota than healthy persons. Previous studies, based on sequencing of V2 region of 16S rRNA gene [26] and quantitative metagenomics [27] have also shown a trend towards reduction of bacterial diversity and of bacterial gene richness, respectively, in patients with cirrhosis from other geographic regions. Reduced microbial diversity has also been reported in patients with many diseases, including Crohn’s disease [28], obesity, insulin resistance and dyslipidemia [29]. The mechanism underlying this reduced diversity of gut bacteria in human disease remains unknown. It is possible that this reduced bacterial diversity is associated with the absence of some specific bacteria, producing a metabolic imbalance due to the unopposed action of the other bacteria.

Despite a difference in the overall composition of gut microbiota in patients with cirrhosis compared to healthy persons, the most abundant phyla in the two group were similar, i.e. Bacteroidetes, Firmicutes and Proteobacteria. Results of the previous studies on this subject have been conflicting. In two of the three previous studies from China, with 98 and 36 patients, respectively, the abundance of Bacteroidetes was reduced, and those of Proteobacteria and Fusobacteria were increased in cirrhosis [27, 30], whereas the third study with 26 patients showed no difference in phylum-level abundances [26]. At lower taxonomic levels, we found that abundances of several bacterial families and genera differed in cirrhosis patients from those in healthy persons, as has been reported previously [31]. However, the bacterial taxa showing such difference in our study were different from those reported in the previous Chinese studies. These differences between studies could be related to differences in several factors, e.g. (i) prevalent microbiota in healthy Chinese and Indian population; (ii) cause of liver disease, with hepatitis B being commoner in China; (iii) dietary habits in the two countries; (iv) severity of liver disease; and, (v) techniques for specimen processing and data analysis between studies.

In particular, we found that abundances of the bacterial groups which are involved in nitrogen metabolism and possess the capability to produce ammonia did not show any difference between the patients and controls. Such a comparison has not been reported previously. This finding suggests that even though the gut microbiota in patients with cirrhosis differs from that in healthy persons, this alteration may not impact the production of nitrogenous substances, which may play a role in causation of HE.

Lactulose is extensively used for the treatment of HE in several parts of the world. On reaching the colon, lactulose is broken down by colonic bacteria (primarily bifidobacteria, lactobacilli and streptococci) into lactic acid, acetic acid and other short-chain fatty acids. Several mechanisms have been proposed for the beneficial effects of lactulose in HE, including (i) its laxative action which reduces the contact time between luminal contents and the intestinal mucosa, reducing the absorption of ammonia; (ii) creation of an acidic environment in the colonic lumen, which traps ammonia by enhancing its conversion into polar and less-absorbable ammonium ions; and, (iii) change in the composition of colonic microbiota, with reduced density of bacteria that produce ammonia and an increased density of those that utilize ammonia for their metabolism [32, 33].

Data on the effect of lactulose on gut microbiota are quite scanty and conflicting [8, 34, 35]. In previous studies, lactulose was shown to facilitate the growth of acidophilic, urease-deficient bacteria, such as lactobacilli and bifidobacteria, in the colon [8, 36]. Another study reported an increase in the number of anaerobic lactobacilli and a decrease in that of Bacteroides spp. after lactulose [37]. However, in yet another study, no association was found between clinical improvement following lactulose and reduction in the number of ammonia-producing bacteria [9]. However, all these studies were based on stool culture, a technique with several inherent limitations, such as the failure of a large majority of colonic bacteria to grow in vitro, and an inability to reliably distinguish between various bacterial groups or to provide a quantitative measure of the relative abundance of various species in a bacterial mixture.

High-throughput 16S rRNA sequencing has a major advantage in that it not only permits accurate species-level identification of various bacterial species present in a complex mixture, but also estimates their relative abundances. This technique has not previously been used to assess the effect of lactulose on intestinal microbiota. Our study, using this technique, failed to show any change in gut microbiota after lactulose administration. This lack of effect was found on analysis at different levels of phylogenetic organization, i.e. from phylum to species level. Further, we also did not find any change in the alpha diversity of the gut microbiota, or of the abundance of bacteria that can produce ammonia. This indicates that the beneficial effect of lactulose on HE may not be related to a change in the composition of gut microbiota, and may instead be mediated by another mechanism. Our findings support a recent report in which Bajaj et al. [38] found no major change, except for a reduction in the abundance in Faecalibacterium spp. (from 6% to 1%), in gut microbiota 14 days after lactulose withdrawal in seven patients with cirrhosis. In our study, bacteria belonging to this genus had an abundance of ~0.1% in both pre- and post-lactulose specimens, with no change after lactulose. Furthermore, we observed a closer similarity of paired (before and after lactulose) specimens from each individual with each other than with specimens from other patients collected at a similar time point; this too supports the conclusion that lactulose did not have a major effect on gut microbiota.

In a recent randomized controlled study, Rahimi et al. compared the effect of lactulose on HE with that of polyethylene glycol, which has a laxative effect but is not expected to alter the gut microbiota. They found the two treatments to be equally effective. This is in consonance with our finding that the effect of lactulose is not mediated by a modulation of gut microbiota and may be related simply to its laxative action [39].

Our data have some limitations. First, we used faecal specimens to study the gut microbiota. Bacterial composition of faeces may differ somewhat from luminal contents of the colon, particularly in the proximal colon, where most of the ammonia or other toxic substances may be produced [40]. However, sampling the colonic luminal contents, e.g., using an endoscope, requires prior cleansing of the gut, which would disrupt the luminal microbiota per se. Second, the diet of patients with cirrhosis may differ from that in healthy persons; this may by itself influence the gut microbiota [41]. Also, alcohol consumption is known to alter gut microbiota [42]. To obviate this, we excluded persons with recent alcohol intake from our study. Third, our study included patients with liver disease of varied causes and severity; this variability may have limited its ability to detect differences between patients with liver cirrhosis and controls. To obviate this problem, it may be useful in the future studies to include a more homogeneous patient group. And, finally, it may be argued that the number of patients in whom we studied the effect of lactulose on gut microbiota was small. However, this component of our study had a paired design, in which each patient serves as his own control, with a higher sensitivity for detecting even minor changes. Also, on a positive note, we ensured that the dose and duration of lactulose administration were adequate.

It would have been interesting to compare the gut flora in patients developing HE despite lactulose treatment versus those who did not develop this complication. However, this was not possible since we did not encounter HE in any of our patients receiving lactulose.

Finally, our data do not rule out the possibility that lactulose may affect the balance of production and utilization of ammonia without changing the species composition of gut microbiota. For instance, lactulose or one of its breakdown products could alter the expression or activity of enzymes in one or more bacterial species without affecting their density or number. The 16S rRNA gene sequencing technique which we used is not able to pick up such changes. This aspect may be studied further in future studies using either shot-gun sequencing of gut bacterial transcriptome or using a metabolomic analysis of fecal water or urine.

## Conclusion

In conclusion, our data indicate that intestinal bacterial microbiota in patients with cirrhosis is different from that in healthy persons; however, whether these changes are primary or are a consequence of liver disease remains unclear. Lactulose administration does not lead to any change in the nature and relative abundance of various bacteria resident in human colon. This suggests that the effect of this drug on HE is possibly mediated by mechanisms other than a change in the composition of gut microbiota.

## Additional files

Additional file 1: Table S1. Comparison of cirrhosis patients who provided paired stool specimens and those who did not provide a post-lactulose specimen. Numerical data are expressed as median (range) and have been compared using Mann-Whitney U test; categorical data are expressed as numbers or proportions and have been compared using Fisher’s exact test. (DOC 36 kb)

Additional file 2: Fig. S1. Results of rarefaction analysis of 16S rRNA sequence reads from 74 stool specimens included in the study. OTUs, operational taxonomic units; the color of each line represents the source of the corresponding specimen. (TIFF 803 kb)

Additional file 3: Table S2. Measures of alpha diversity for gut flora in stool specimens from patients with liver cirrhosis and healthy controls. (DOC 118 kb)

Additional file 4: Table S3. Comparison of abundances of various bacterial groups at different levels of taxonomy (phyla, classes, orders, genera and species). The rows for groups showing significant differences (after Benjamini-Hochberg correction) are shaded pink. (DOC 289 kb)

Additional file 5: Figure S2. Comparison of abundances of different bacteria in gut microbiota, between healthy controls and liver cirrhosis, at the level of class (a), order (b) and genus (c). Data are shown using box-plots and percent values on a log10 scale. Asterix marks and italic font point to the groups where the values for patients and controls showed significant differences. (TIFF 4056 kb)

Additional file 6: Figure S3. Comparison of abundances of gut microbiome bacteria, in patients with liver cirrhosis before and after 6 weeks of lactulose use, at the level of (a) class (b) order and (c) genus on a log10 scale. None of the bacterial groups showed any significant difference. (TIFF 6332 kb)

## Abbreviations

CPT: Child-Turcotte-Pugh
HE: Hepatic encephalopathy
PCoA: Principal co-ordinate analysis
