# Supplementary material for: Faecal bacterial microbiota in patients with cirrhosis and the effect of lactulose administration
Source: BMC Gastroenterol. 2017 Nov 28;17:125. doi: 10.1186/s12876-017-0683-9 (PMC5704526; doi:10.1186/s12876-017-0683-9)
Supplement: Supplementary file 1 — Comparison of cirrhosis patients who provided paired stool specimens and those who did not provide a post-lactulose specimen. Numerical data are expressed as median (range) and have been compared using Mann-Whitney U test; categorical data are expressed as numbers or proportions and have been compared using Fisher’s exact test. (DOC 36 kb) [file 12876_2017_683_MOESM1_ESM.doc]

**Table S1. Comparison of cirrhosis patients who provided paired stool specimens and those who did not provide a post-lactulose specimen**

| **Parameter** | **Group with paired specimens (n=21)** | **Group with unpaired specimens (n=14)** | **p value** |
| --- | --- | --- | --- |
| Age (y) | 45 (29-64) | 40 (30-65) | 0.38 |
| Gender (M/F) | 13/8 | 13/1 | 0.06 |
| Body mass index (Kg/m^2^) | 22.5 (18.8-32.3) | 22.8 (17.3-29.4) | 0.61 |
| Cause of liver disease  Alcohol  Hepatitis B virus  Hepatitis C virus  Autoimmune hepatitis  Cryptogenic | 2  4  6  1  8 | 5  3  3  0  3 | 0.34 |
| Child-Turcotte-Pugh class  A  B  C | 7  13  1 | 3  8  3 | 0.29 |
| *Complications of portal hypertension* | | | |
| Ascites | 10 | 9 | 0.33 |
| Spontaneous bacterial peritonitis | 2 | 4 | 0.19 |
| Hepatic encephalopathy | 2 | 2 | 1.00 |
| Variceal bleed | 2 | 6 | 0.04 |
| *Disease severity scores* | | | |
| Child-Turcotte-Pugh score | 7 (5-10) | 8 (5-11) | 0.19 |
| Model for end-stage liver disease (MELD) score | 11 (6-25) | 14 (6-22) | 0.21 |

Numerical data are expressed as median (range) and have been compared using Mann-Whitney U test; categorical data are expressed as numbers or proportions and have been compared using Fisher’s exact test
