# Supplementary figures and images for: Faecal bacterial microbiota in patients with cirrhosis and the effect of lactulose administration
Source: BMC Gastroenterol. 2017 Nov 28;17:125. doi: 10.1186/s12876-017-0683-9 (PMC5704526; doi:10.1186/s12876-017-0683-9)

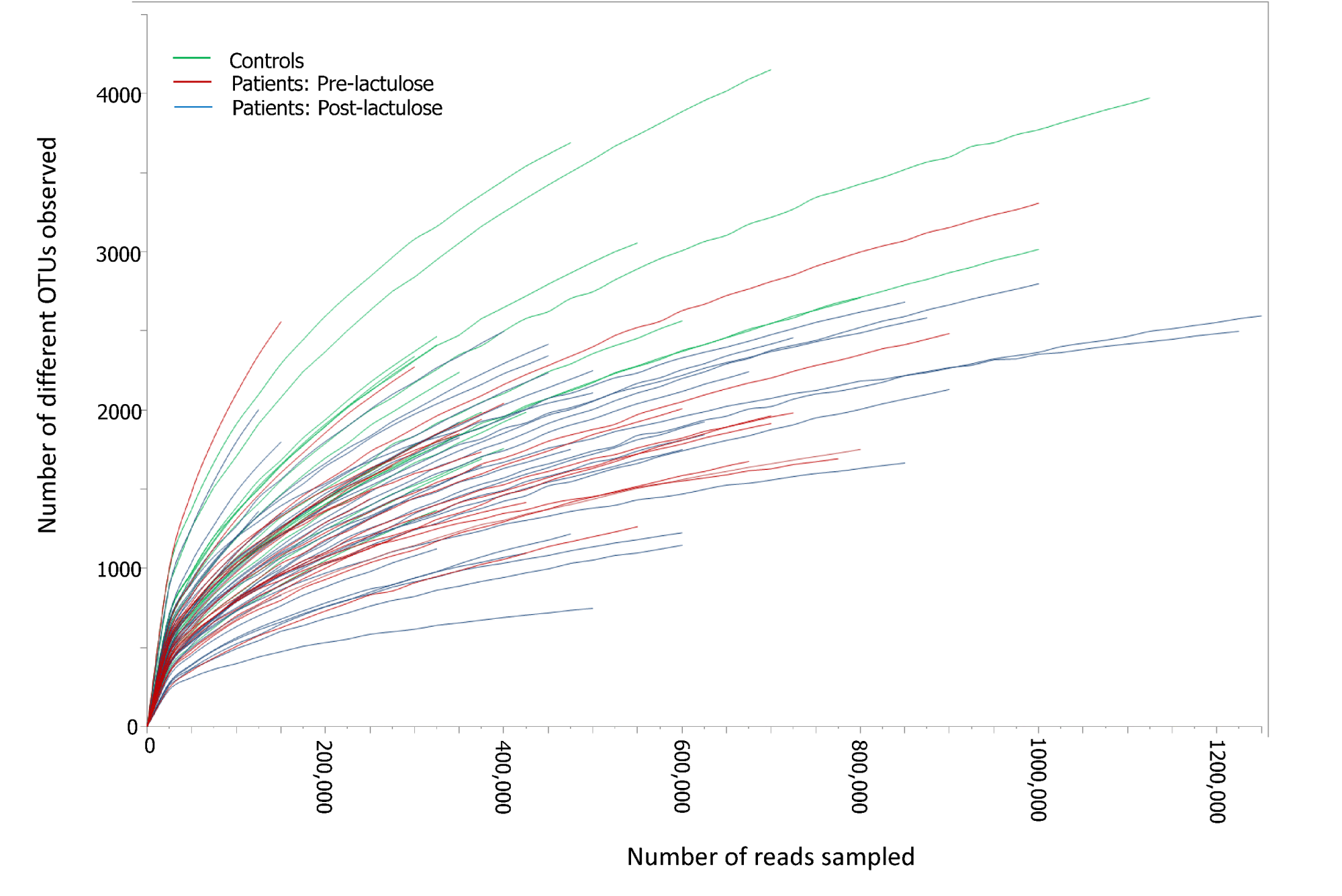

Supplement: Supplementary file 2 — Results of rarefaction analysis of 16S rRNA sequence reads from 74 stool specimens included in the study. OTUs, operational taxonomic units; the color of each line represents the source of the corresponding specimen. (TIFF 803 kb) [file 12876_2017_683_MOESM2_ESM.tif]

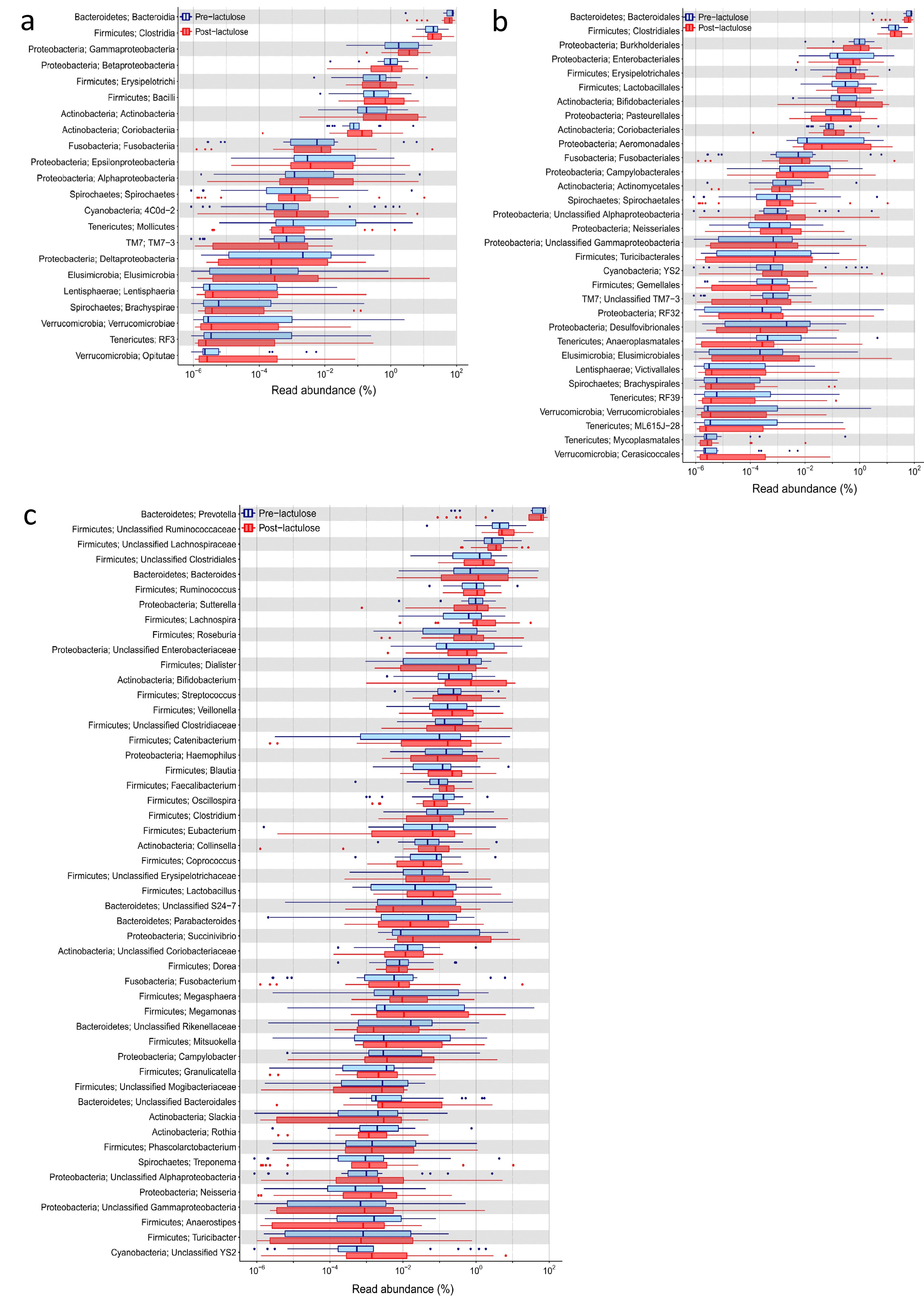

Supplement: Supplementary file 6 — Comparison of abundances of gut microbiome bacteria, in patients with liver cirrhosis before and after 6 weeks of lactulose use, at the level of (a) class (b) order and (c) genus on a log10 scale. None of the bacterial groups showed any significant difference. (TIFF 6332 kb) [file 12876_2017_683_MOESM6_ESM.tif]
