# Supplementary material for: Faecal bacterial microbiota in patients with cirrhosis and the effect of lactulose administration
Source: BMC Gastroenterol. 2017 Nov 28;17:125. doi: 10.1186/s12876-017-0683-9 (PMC5704526; doi:10.1186/s12876-017-0683-9)
Supplement: Supplementary file 3 — Measures of alpha diversity for gut flora in stool specimens from patients with liver cirrhosis and healthy controls. (DOC 118 kb) [file 12876_2017_683_MOESM3_ESM.doc]

**Table S2. Measures of alpha diversity for gut flora in stool specimens from patients with liver cirrhosis and healthy controls**

| Subject ID | Group | Alpha diversity | | | | | | | | | |
| --- | --- | --- | --- | --- | --- | --- | --- | --- | --- | --- | --- |
|  |  | Baseline | | | | | After lactulose | | | | |
|  |  | Observed | ACE | Chao1 | Shannon | Simpson | Observed | ACE | Chao1 | Shannon | Simpson |
| 002H | Healthy | 4163 | 6984 | 6790 | 6.6 | 0.97 |  |  |  |  |  |
| 003H | Healthy | 3108 | 5450 | 5004 | 6.0 | 0.96 |  |  |  |  |  |
| 004H | Healthy | 2603 | 4834 | 4698 | 5.6 | 0.95 |  |  |  |  |  |
| 005H | Healthy | 2709 | 5018 | 4777 | 5.6 | 0.95 |  |  |  |  |  |
| 006H | Healthy | 3027 | 5583 | 5679 | 5.3 | 0.94 |  |  |  |  |  |
| 007H | Healthy | 3749 | 5709 | 5569 | 6.4 | 0.96 |  |  |  |  |  |
| 008H | Healthy | 3991 | 6500 | 6264 | 5.9 | 0.96 |  |  |  |  |  |
| 009H | Healthy | 2039 | 4138 | 3983 | 5.6 | 0.95 |  |  |  |  |  |
| 010H | Healthy | 2024 | 4305 | 4464 | 5.7 | 0.96 |  |  |  |  |  |
| 011H | Healthy | 2396 | 4726 | 4484 | 6.5 | 0.97 |  |  |  |  |  |
| 012H | Healthy | 2245 | 4371 | 4299 | 5.5 | 0.93 |  |  |  |  |  |
| 013H | Healthy | 1788 | 3399 | 3325 | 4.4 | 0.90 |  |  |  |  |  |
| 014H | Healthy | 1735 | 3793 | 3511 | 4.8 | 0.93 |  |  |  |  |  |
| 016H | Healthy | 2417 | 4770 | 4588 | 6.4 | 0.96 |  |  |  |  |  |
| 018H | Healthy | 2480 | 4659 | 4622 | 6.4 | 0.97 |  |  |  |  |  |
| 021H | Healthy | 1417 | 3423 | 3063 | 4.8 | 0.92 |  |  |  |  |  |
| 022H | Healthy | 1943 | 3394 | 3116 | 4.8 | 0.87 |  |  |  |  |  |
| 023H | Healthy | 1840 | 3814 | 3437 | 5.6 | 0.95 |  |  |  |  |  |
| 101A | Cirrhosis | 1940 | 3733 | 3522 | 4.9 | 0.94 | 3338 | 5783 | 5598 | 5.4 | 0.94 |
| 102A | Cirrhosis | 2683 | 4339 | 4127 | 5.1 | 0.92 |  |  |  |  |  |
| 103A | Cirrhosis | 2819 | 5165 | 4731 | 5.1 | 0.93 | 2496 | 4642 | 4694 | 5.0 | 0.92 |
| 104A | Cirrhosis | 1453 | 3378 | 3174 | 4.9 | 0.92 | 1998 | 4350 | 4000 | 4.9 | 0.94 |
| 105A | Cirrhosis | 2152 | 4397 | 4351 | 5.0 | 0.94 |  |  |  |  |  |
| 106A | Cirrhosis | 1668 | 2387 | 2377 | 3.8 | 0.80 |  |  |  |  |  |
| 107A | Cirrhosis | 2268 | 4295 | 4170 | 5.9 | 0.96 | 1525 | 2612 | 2623 | 5.6 | 0.93 |
| 108A | Cirrhosis | 1242 | 3006 | 2819 | 4.6 | 0.94 | 1767 | 2769 | 2597 | 3.9 | 0.86 |

| Subject ID | Group | Alpha diversity | | | | | | | | | |
| --- | --- | --- | --- | --- | --- | --- | --- | --- | --- | --- | --- |
|  |  | Baseline | | | | | After lactulose | | | | |
|  |  | Observed | ACE | Chao1 | Shannon | Simpson | Observed | ACE | Chao1 | Shannon | Simpson |
| 109A | Cirrhosis | 1124 | 2284 | 2441 | 4.4 | 0.91 | 2005 | 3233 | 3139 | 5.0 | 0.95 |
| 111A | Cirrhosis | 1166 | 2124 | 2071 | 3.8 | 0.87 |  |  |  |  |  |
| 112A | Cirrhosis | 1877 | 3228 | 3157 | 3.9 | 0.81 | 1943 | 3480 | 3647 | 4.5 | 0.87 |
| 113A | Cirrhosis | 1125 | 1936 | 1911 | 4.0 | 0.87 | 2033 | 3845 | 3685 | 5.5 | 0.96 |
| 114A | Cirrhosis | 883 | 1544 | 1474 | 5.2 | 0.94 | 1691 | 3206 | 3142 | 3.8 | 0.83 |
| 115A | Cirrhosis | 1231 | 1900 | 1895 | 3.2 | 0.79 | 1693 | 2387 | 2332 | 4.4 | 0.90 |
| 116A | Cirrhosis | 1762 | 2908 | 2904 | 4.3 | 0.89 | 1188 | 2078 | 2013 | 4.0 | 0.83 |
| 117A | Cirrhosis | 2286 | 4020 | 3925 | 5.1 | 0.93 | 1289 | 2839 | 2741 | 4.1 | 0.90 |
| 118A | Cirrhosis | 2610 | 4074 | 3806 | 4.5 | 0.91 |  |  |  |  |  |
| 119A | Cirrhosis | 2374 | 3952 | 3840 | 5.0 | 0.90 |  |  |  |  |  |
| 120A | Cirrhosis | 2498 | 3249 | 3071 | 4.9 | 0.91 | 1440 | 2113 | 2138 | 4.4 | 0.85 |
| 121A | Cirrhosis | 2582 | 4013 | 3904 | 5.5 | 0.95 |  |  |  |  |  |
| 122A | Cirrhosis | 2273 | 4136 | 4090 | 5.3 | 0.94 |  |  |  |  |  |
| 123A | Cirrhosis | 1754 | 2938 | 2978 | 4.3 | 0.88 |  |  |  |  |  |
| 124A | Cirrhosis | 2481 | 4328 | 4268 | 5.2 | 0.94 |  |  |  |  |  |
| 125A | Cirrhosis | 1027 | 2671 | 2328 | 4.2 | 0.90 |  |  |  |  |  |
| 126A | Cirrhosis | 750 | 1081 | 1073 | 3.4 | 0.84 |  |  |  |  |  |
| 127A | Cirrhosis | 2112 | 2882 | 2904 | 5.9 | 0.96 | 1472 | 2837 | 2821 | 5.1 | 0.94 |
| 129A | Cirrhosis | 1837 | 3232 | 3002 | 5.3 | 0.93 |  |  |  |  |  |
| 130A | Cirrhosis | 1358 | 2615 | 2316 | 4.0 | 0.82 | 1858 | 3468 | 3450 | 4.9 | 0.92 |
| 131B | Cirrhosis | 1861 | 2824 | 2834 | 5.6 | 0.96 |  |  |  |  |  |
| 132A | Cirrhosis | 2433 | 4237 | 4455 | 5.5 | 0.95 | 1691 | 2792 | 2767 | 5.2 | 0.94 |
| 133A | Cirrhosis | 2027 | 3985 | 3642 | 6.4 | 0.96 | 2588 | 4279 | 3827 | 6.6 | 0.97 |
| 134A | Cirrhosis | 1653 | 4012 | 3836 | 5.4 | 0.95 | 2309 | 4505 | 4203 | 6.2 | 0.97 |
| 136A | Cirrhosis | 1503 | 3434 | 3122 | 5.4 | 0.95 | 1766 | 2665 | 2621 | 4.9 | 0.92 |
| 137A | Cirrhosis | 2538 | 4665 | 4545 | 6.1 | 0.96 | 2098 | 4051 | 3786 | 4.9 | 0.92 |
| 138A | Cirrhosis | 1988 | 3963 | 3890 | 4.8 | 0.89 | 1949 | 3921 | 3700 | 5.3 | 0.95 |

ACE, abundance-based coverage estimator.
Of 31 patients with cirrhosis, follow-up specimens (after lactulose administration) were available for only 17.
