# Supplementary material for: Faecal bacterial microbiota in patients with cirrhosis and the effect of lactulose administration
Source: BMC Gastroenterol. 2017 Nov 28;17:125. doi: 10.1186/s12876-017-0683-9 (PMC5704526; doi:10.1186/s12876-017-0683-9)
Supplement: Supplementary file 4 — Comparison of abundances of various bacterial groups at different levels of taxonomy (phyla, classes, orders, genera and species). The rows for groups showing significant differences (after Benjamini-Hochberg correction) are shaded pink. (DOC 289 kb) [file 12876_2017_683_MOESM4_ESM.doc]

**Table S3. Comparison of abundances of various bacterial groups at different levels of taxonomy (phyla, classes, orders, genera and species)**. The rows for groups showing significant differences (after Benjamini-Hochberg correction) are shaded pink.

| **Taxonomic group** | **P value (FDR)** | **Controls** | | **Cirrhosis** | |
| --- | --- | --- | --- | --- | --- |
|  |  | **Median** | **[Range]** | **Median** | **[Range]** |
| **Phylum** | | | | | |
| Bacteroidetes | 0.62 | 66.82 | [30.35-88.99] | 71.91 | [0.11-90.01] |
| Firmicutes | 0.62 | 18.65 | [3.95-43.47] | 21.95 | [6.95-74.56] |
| Proteobacteria | 0.05 | 8.2 | [1.34-48.35] | 4.37 | [0.61-50.64] |
| Cyanobacteria | 7.80E-04 | 0.53 | [0-5.46] | 2.00E-04 | [0-1.82] |
| Actinobacteria | 0.66 | 0.44 | [0.07-4.64] | 0.35 | [0.02-6.76] |
| Tenericutes | 1.10E-03 | 0.07 | [4.90E-04-1.03] | 8.40E-04 | [0-4.97] |
| Lentisphaerae | 6.50E-04 | 0.01 | [0-0.2] | 0 | [0-0.02] |
| Spirochaetes | 0.04 | 1.40E-03 | [0-7.62] | 6.50E-04 | [0-4.44] |
| Elusimicrobia | 0.01 | 1.30E-03 | [0-0.13] | 0 | [0-0.84] |
| Fusobacteria | 0.24 | 8.40E-04 | [0-0.1] | 4.00E-03 | [0-13.74] |
| Verrucomicrobia | 0.62 | 3.40E-04 | [0-0.12] | 2.00E-04 | [0-2.59] |
|  | | | | | |
| **Class** | | | | | |
| p_Bacteroidetes; c_Bacteroidia | 0.66 | 66.82 | [30.35-88.99] | 71.91 | [0.11-90.01] |
| p_Firmicutes; c_Clostridia | 0.73 | 16.4 | [3.51-41.76] | 20.82 | [6.04-57.92] |
| p_Proteobacteria; c_Gammaproteobacteria | 0.09 | 6.5 | [0.11-40.77] | 1.75 | [0.04-50.6] |
| p_Firmicutes; c_Erysipelotrichi | 0.01 | 1.57 | [0.29-7.23] | 0.44 | [0-12.42] |
| p_Proteobacteria; c_Betaproteobacteria | 0.93 | 0.72 | [0.31-4.83] | 0.91 | [0-3.48] |
| p_Cyanobacteria; c_4C0d-2 | 1.50E-03 | 0.53 | [0-5.46] | 0 | [0-1.82] |
| p_Firmicutes; c_Bacilli | 0.63 | 0.31 | [0.05-2.3] | 0.35 | [0.01-53.42] |
| p_Actinobacteria; c_Actinobacteria | 0.66 | 0.21 | [0.01-3.94] | 0.23 | [5.00E-03-3.37] |
| p_Actinobacteria; c_Coriobacteriia | 0.24 | 0.1 | [0.03-0.7] | 0.07 | [2.00E-04-4.84] |
| p_Tenericutes; c_Mollicutes | 4.20E-03 | 0.05 | [4.90E-04-0.98] | 6.70E-04 | [0-4.72] |
| p_Proteobacteria; c_Deltaproteobacteria | 0.09 | 0.02 | [4.90E-04-0.1] | 2.80E-03 | [0-0.32] |
| p_Proteobacteria; c_Alphaproteobacteria | 0.01 | 0.01 | [0-8.33] | 9.60E-04 | [0-7.63] |
| p_Lentisphaerae; c_Lentisphaeria | 1.20E-03 | 0.01 | [0-0.2] | 0 | [0-0.02] |
| p_Proteobacteria; c_Epsilonproteobacteria | 0.93 | 2.70E-03 | [0-1.69] | 3.10E-03 | [0-1.84] |
| p_Elusimicrobia; c_Elusimicrobia | 0.01 | 1.30E-03 | [0-0.13] | 0 | [0-0.84] |
| p_Spirochaetes; c_Spirochaetes | 0.11 | 1.10E-03 | [0-7.23] | 6.50E-04 | [0-4.29] |
| p_Fusobacteria; c_Fusobacteriia | 0.24 | 8.40E-04 | [0-0.1] | 4.10E-03 | [0-13.74] |
| p_Tenericutes; c_RF3 | 0.01 | 5.60E-04 | [0-0.71] | 0 | [0-0.26] |
| p_Spirochaetes; c_Brachyspirae | 0.25 | 0 | [0-0.39] | 0 | [0-0.15] |
| p_Verrucomicrobia; c_Opitutae | 0.06 | 0 | [0-0.12] | 0 | [0-0.02] |
| p_Verrucomicrobia; c_Verrucomicrobiae | 0.66 | 0 | [0-0.04] | 0 | [0-2.59] |
|  | | | | | |
| **Order** | | | | | |
| p_Bacteroidetes; c_Bacteroidia; o_Bacteroidales | 0.65 | 66.82 | [30.35-88.99] | 71.91 | [0.11-90.01] |
| p_Firmicutes; c_Clostridia; o_Clostridiales | 0.73 | 16.4 | [3.51-41.76] | 20.82 | [6.04-57.92] |
| p_Proteobacteria; c_Gammaproteobacteria; o_Aeromonadales | 1.30E-05 | 5.99 | [0.03-40.63] | 0.01 | [0-7.57] |
| p_Firmicutes; c_Erysipelotrichi; o_Erysipelotrichales | 0.01 | 1.57 | [0.29-7.23] | 0.44 | [1.40E-03-12.42] |
| p_Proteobacteria; c_Betaproteobacteria; o_Burkholderiales | 0.77 | 0.72 | [0.31-4.83] | 0.91 | [3.30E-03-3.48] |
| p_Cyanobacteria; c_4C0d-2; o_YS2 | 1.10E-03 | 0.53 | [0-5.46] | 2.00E-04 | [0-1.82] |
| p_Firmicutes; c_Bacilli; o_Lactobacillales | 0.49 | 0.29 | [0.03-1.2] | 0.35 | [0.01-53.37] |
| p_Proteobacteria; c_Gammaproteobacteria; o_Enterobacteriales | 0.65 | 0.24 | [0.02-7.66] | 0.47 | [0.01-28.28] |
| p_Actinobacteria; c_Actinobacteria; o_Bifidobacteriales | 0.74 | 0.21 | [0.01-3.94] | 0.23 | [3.60E-03-3.36] |
| p_Actinobacteria; c_Coriobacteriia; o_Coriobacteriales | 0.21 | 0.1 | [0.03-0.7] | 0.07 | [2.00E-04-4.84] |
| p_Tenericutes; c_Mollicutes; o_RF39 | 5.90E-04 | 0.04 | [2.00E-04-0.98] | 1.70E-04 | [0-0.29] |
| p_Proteobacteria; c_Gammaproteobacteria; o_Pasteurellales | 1.20E-03 | 0.04 | [1.40E-03-0.37] | 0.27 | [0.01-22.33] |
| p_Proteobacteria; c_Deltaproteobacteria; o_Desulfovibrionales | 0.08 | 0.02 | [4.90E-04-0.1] | 2.80E-03 | [0-0.32] |
| p_Firmicutes; c_Bacilli; o_Turicibacterales | 0.01 | 0.01 | [2.90E-04-1.88] | 8.00E-04 | [0-0.18] |
| p_Proteobacteria; c_Alphaproteobacteria; o_UCAlphaproteobacteria | 0.01 | 0.01 | [0-7.92] | 6.20E-04 | [0-2.77] |
| p_Lentisphaerae; c_Lentisphaeria; o_Victivallales | 5.91E-04 | 0.01 | [0-0.2] | 0 | [0-0.02] |
| p_Proteobacteria; c_Epsilonproteobacteria; o_Campylobacterales | 0.90 | 2.70E-03 | [0-1.69] | 3.10E-03 | [0-1.84] |
| p_Elusimicrobia; c_Elusimicrobia; o_Elusimicrobiales | 0.01 | 1.30E-03 | [0-0.13] | 0 | [0-0.84] |
| p_Actinobacteria; c_Actinobacteria; o_Actinomycetales | 0.05 | 1.30E-03 | [0-0.01] | 4.00E-03 | [0-0.7] |
| p_Spirochaetes; c_Spirochaetes; o_Spirochaetales | 0.10 | 1.10E-03 | [0-7.23] | 6.50E-04 | [0-4.29] |
| p_Tenericutes; c_Mollicutes; o_Anaeroplasmatales | 0.06 | 1.10E-03 | [0-0.3] | 4.20E-04 | [0-4.72] |
| p_Proteobacteria; c_Alphaproteobacteria; o_RF32 | 0.01 | 0 | [0-1.25] | 0 | [0-7.62] |
| p_Fusobacteria; c_Fusobacteriia; o_Fusobacteriales | 0.21 | 8.40E-04 | [0-0.1] | 4.10E-03 | [0-13.74] |
| p_Tenericutes; c_RF3; o_ML615J-28 | 0.01 | 5.60E-04 | [0-0.71] | 0 | [0-0.26] |
| p_Proteobacteria; c_Betaproteobacteria; o_Neisseriales | 0.08 | 5.00E-05 | [0-0.01] | 8.10E-04 | [0-0.57] |
| p_Spirochaetes; c_Brachyspirae; o_Brachyspirales | 0.23 | 0 | [0-0.39] | 0 | [0-0.15] |
| p_Verrucomicrobia; c_Opitutae; o_Cerasicoccales | 0.05 | 0 | [0-0.12] | 0 | [0-0.02] |
| p_Firmicutes; c_Bacilli; o_Gemellales | 0.11 | 0 | [0-1.90E-03] | 1.70E-04 | [0-0.04] |
| p_Proteobacteria; c_Gammaproteobacteria; o_Unc; Gammaproteobacteria | 0.08 | 0 | [0-0.79] | 2.00E-04 | [0-0.51] |
| p_Verrucomicrobia; c_Verrucomicrobiae; o_Verrucomicrobiales | 0.65 | 0 | [0-0.04] | 0 | [0-2.59] |
|  |  |  |  |  |  |
| **Family** |  |  |  |  |  |
| p_Bacteroidetes; c_Bacteroidia; o_Bacteroidales; f_Prevotellaceae | 0.93 | 59.53 | [10.72-85.79] | 60.98 | [0.07-87.3] |
| p_Proteobacteria; c_Gammaproteobacteria; o_Aeromonadales; f_Succinivibrionaceae | 2.30E-05 | 5.99 | [0.03-40.63] | 0.01 | [0-7.57] |
| p_Firmicutes; c_Clostridia; o_Clostridiales; f_Lachnospiraceae | 0.48 | 5.74 | [1.52-10.79] | 7.55 | [1.07-36.22] |
| p_Firmicutes; c_Clostridia; o_Clostridiales; f_Ruminococcaceae | 0.69 | 5.02 | [1.18-17.95] | 5.04 | [0.02-27.37] |
| p_Bacteroidetes; c_Bacteroidia; o_Bacteroidales; f_Paraprevotellaceae | 0.11 | 3.08 | [0.1-12.14] | 0.86 | [2.20E-03-13.73] |
| p_Firmicutes; c_Clostridia; o_Clostridiales; f_UncClostridiales | 0.06 | 1.79 | [0.35-4.59] | 0.66 | [3.10E-03-7.17] |
| p_Firmicutes; c_Erysipelotrichi; o_Erysipelotrichales; f_Erysipelotrichaceae | 0.01 | 1.57 | [0.29-7.23] | 0.44 | [1.40E-03-12.42] |
| p_Firmicutes; c_Clostridia; o_Clostridiales; f_Veillonellaceae | 0.34 | 1.33 | [0.33-13.51] | 2.95 | [0.17-44.68] |
| p_Proteobacteria; c_Betaproteobacteria; o_Burkholderiales; f_Alcaligenaceae | 0.85 | 0.72 | [0.3-4.83] | 0.83 | [3.30E-03-3.47] |
| p_Bacteroidetes; c_Bacteroidia; o_Bacteroidales; f_UncBacteroidales | 3.50E-03 | 0.63 | [7.40E-04-7.55] | 1.70E-03 | [0-1.97] |
| p_Cyanobacteria; c_4C0d-2; o_YS2; f_Unc YS2 | 2.00E-03 | 0.53 | [0-5.46] | 2.00E-04 | [0-1.82] |
| p_Firmicutes; c_Clostridia; o_Clostridiales; f_UncCoriobacteriales | 0.23 | 0.37 | [0.08-1.36] | 0.15 | [4.10E-04-3.7] |
| p_Bacteroidetes; c_Bacteroidia; o_Bacteroidales; f_S24-7 | 0.11 | 0.28 | [2.30E-03-2] | 0.03 | [0-11.51] |
| p_Bacteroidetes; c_Bacteroidia; o_Bacteroidales; f_Bacteroidaceae | 0.07 | 0.27 | [0.07-14.13] | 1.19 | [0.01-55.27] |
| p_Proteobacteria; c_Gammaproteobacteria; o_Enterobacteriales; f_Enterobacteriaceae | 0.60 | 0.24 | [0.02-7.66] | 0.47 | [0.01-28.28] |
| p_Actinobacteria; c_Actinobacteria; o_Bifidobacteriales; f_Bifidobacteriaceae | 0.74 | 0.21 | [0.01-3.94] | 0.23 | [3.60E-03-3.36] |
| p_Firmicutes; c_Clostridia; o_Clostridiales; f_Clostridiaceae | 0.31 | 0.16 | [0.03-1.36] | 0.31 | [4.10E-04-3.43] |
| p_Firmicutes; c_Bacilli; o_Lactobacillales; f_Lactobacillaceae | 0.23 | 0.11 | [2.50E-03-1.1] | 0.03 | [1.70E-04-2.84] |
| p_Actinobacteria; c_Coriobacteriia; o_Coriobacteriales; f_Coriobacteriaceae | 0.22 | 0.1 | [0.03-0.7] | 0.07 | [2.00E-04-4.84] |
| p_Firmicutes; c_Bacilli; o_Lactobacillales; f_Streptococcaceae | 0.11 | 0.1 | [0.01-0.69] | 0.26 | [0.01-53] |
| p_Tenericutes; c_Mollicutes; o_RF39; f_Unc RF39 | 1.10E-03 | 0.04 | [2.00E-04-0.98] | 1.70E-04 | [0-0.29] |
| p_Proteobacteria; c_Gammaproteobacteria; o_Pasteurellales; f_Pasteurellaceae | 2.20E-03 | 0.04 | [1.40E-03-0.37] | 0.27 | [0.01-22.33] |
| p_Bacteroidetes; c_Bacteroidia; o_Bacteroidales; f_Porphyromonadaceae | 0.99 | 0.03 | [1.50E-03-0.87] | 0.05 | [0-1.37] |
| p_Bacteroidetes; c_Bacteroidia; o_Bacteroidales; f_Rikenellaceae | 0.48 | 0.03 | [1.30E-03-0.8] | 0.02 | [0-1.21] |
| p_Proteobacteria; c_Deltaproteobacteria; o_Desulfovibrionales; f_Desulfovibrionaceae | 0.09 | 0.02 | [4.90E-04-0.1] | 2.80E-03 | [0-0.32] |
| p_Bacteroidetes; c_Bacteroidia; o_Bacteroidales; f_Odoribacteraceae | 0.15 | 0.02 | [0-0.44] | 1.00E-03 | [0-0.43] |
| p_Firmicutes; c_Bacilli; o_Turicibacterales; f_Turicibacteraceae | 0.02 | 0.01 | [2.90E-04-1.88] | 8.00E-04 | [0-0.18] |
| p_Firmicutes; c_Clostridia; o_Clostridiales; f_Christensenellaceae | 0.06 | 0.01 | [0-0.41] | 8.40E-05 | [0-0.16] |
| p_Firmicutes; c_Clostridia; o_Clostridiales; f_Mogibacteriaceae | 0.07 | 0.01 | [1.50E-03-0.08] | 3.80E-03 | [0-0.07] |
| p_Proteobacteria; c_Alphaproteobacteria; o_UncAlphaproteobacteria; f_UncAlphaproteobacteria | 0.01 | 0.01 | [0-7.92] | 6.20E-04 | [0-2.77] |
| p_Lentisphaerae; c_Lentisphaeria; o_Victivallales; f_Victivallaceae | 1.10E-03 | 0.01 | [0-0.2] | 0 | [0-0.02] |
| p_Firmicutes; c_Clostridia; o_Clostridiales; f_Peptococcaceae | 0.01 | 3.90E-03 | [0-0.02] | 0 | [0-0.04] |
| p_Proteobacteria; c_Epsilonproteobacteria; o_Campylobacterales; f_Campylobacteraceae | 0.41 | 1.70E-03 | [0-1.11] | 3.10E-03 | [0-1.84] |
| p_Elusimicrobia; c_Elusimicrobia; o_Elusimicrobiales; f_Elusimicrobiaceae | 0.02 | 1.30E-03 | [0-0.13] | 0 | [0-0.84] |
| p_Actinobacteria; c_Actinobacteria; o_Actinomycetales; f_Micrococcaceae | 0.06 | 1.30E-03 | [0-3.40E-03] | 3.40E-03 | [0-0.69] |
| p_Spirochaetes; c_Spirochaetes; o_Spirochaetales; f_Spirochaetaceae | 0.11 | 1.10E-03 | [0-7.23] | 6.50E-04 | [0-4.29] |
| p_Tenericutes; c_Mollicutes; o_Anaeroplasmatales; f_Anaeroplasmataceae | 0.07 | 1.10E-03 | [0-0.3] | 4.20E-04 | [0-4.72] |
| p_Proteobacteria; c_Alphaproteobacteria; o_RF32; f_Unc RF32 | 0.02 | 9.30E-04 | [0-1.25] | 0 | [0-7.62] |
| p_Fusobacteria; c_Fusobacteriia; o_Fusobacteriales; f_Fusobacteriaceae | 0.23 | 8.40E-04 | [0-0.1] | 4.10E-03 | [0-13.74] |
| p_Bacteroidetes; c_Bacteroidia; o_Bacteroidales; f_Barnesiellaceae | 0.26 | 8.20E-04 | [0-3.55] | 2.70E-04 | [0-1.57] |
| p_Firmicutes; c_Bacilli; o_Lactobacillales; f_Carnobacteriaceae | 0.04 | 7.10E-04 | [0-0.01] | 2.90E-03 | [0-0.21] |
| p_Tenericutes; c_RF3; o_ML615J-28; f_Unc ML615J-28 | 0.01 | 5.60E-04 | [0-0.71] | 0 | [0-0.26] |
| p_Firmicutes; c_Bacilli; o_Lactobacillales; f_Leuconostocaceae | 0.65 | 4.00E-04 | [0-0.05] | 2.10E-04 | [0-0.09] |
| p_Firmicutes; c_Bacilli; o_Lactobacillales; f_Enterococcaceae | 0.16 | 2.70E-04 | [0-0.01] | 0 | [0-0.09] |
| p_Actinobacteria; c_Actinobacteria; o_Actinomycetales; f_Actinomycetaceae | 0.30 | 2.30E-04 | [0-2.30E-03] | 2.90E-04 | [0-0.05] |
| p_Proteobacteria; c_Betaproteobacteria; o_Burkholderiales; f_Oxalobacteraceae | 0.08 | 2.00E-04 | [0-0.07] | 0 | [0-0.02] |
| p_Proteobacteria; c_Epsilonproteobacteria; o_Campylobacterales; f_Helicobacteraceae | 0.01 | 1.90E-04 | [0-1.69] | 0 | [0-0.24] |
| p_Proteobacteria; c_Betaproteobacteria; o_Neisseriales; f_Neisseriaceae | 0.09 | 5.00E-05 | [0-0.01] | 8.10E-04 | [0-0.57] |
| p_Spirochaetes; c_Brachyspirae; o_Brachyspirales; f_Brachyspiraceae | 0.24 | 0 | [0-0.39] | 0 | [0-0.15] |
| p_Proteobacteria; c_Betaproteobacteria; o_Burkholderiales; f_Burkholderiaceae | 0.02 | 0 | [0-4.90E-03] | 0 | [0-0.06] |
| p_Verrucomicrobia; c_Opitutae; o_Cerasicoccales; f_Cerasicoccaceae | 0.06 | 0 | [0-0.12] | 0 | [0-0.02] |
| p_Proteobacteria; c_Betaproteobacteria; o_Burkholderiales; f_Comamonadaceae | 0.26 | 0 | [0-0.08] | 0 | [0-0.12] |
| p_Firmicutes; c_Bacilli; o_Gemellales; f_Gemellaceae | 0.12 | 0 | [0-1.90E-03] | 1.70E-04 | [0-0.04] |
| p_Bacteroidetes; c_Bacteroidia; o_Bacteroidales; f_UncBacteroidales | 0.90 | 0 | [0-0.19] | 0 | [0-0.12] |
| p_Proteobacteria; c_Gammaproteobacteria; o_UncGammaproteobacteria; f_UncGammaproteobacteria | 0.09 | 0 | [0-0.79] | 2.00E-04 | [0-0.51] |
| p_Verrucomicrobia; c_Verrucomicrobiae; o_Verrucomicrobiales; f_Verrucomicrobiaceae | 0.63 | 0 | [0-0.04] | 0 | [0-2.59] |
|  | | | | | |
| **Genus** | | | | | |
| p_Bacteroidetes; c_Bacteroidia; o_Bacteroidales; f_Paraprevotellaceae; g_Prevotella | 0.19 | 59.53 | [10.72-85.79] | 60.98 | [0.07-87.3] |
| p_Proteobacteria; c_Gammaproteobacteria; o_Aeromonadales; f_Succinivibrionaceae; g_Succinivibrio | 3.10E-05 | 5.99 | [0.03-40.58] | 0.01 | [0-7.57] |
| p_Firmicutes; c_Clostridia; o_Clostridiales; f_Ruminococcaceae; g_UncRuminococcaceae | 0.05 | 4.1 | [1.07-15.21] | 4.59 | [0.02-23.04] |
| p_Firmicutes; c_Clostridia; o_Clostridiales; f_Lachnospiraceae; g_UncLachnospiraceae | 0.33 | 3.23 | [0.43-7.86] | 2.39 | [0.02-16.76] |
| p_Bacteroidetes; c_Bacteroidia; o_Bacteroidales; f_Prevotellaceae; g_Prevotella | 0.96 | 3.04 | [0.01-12.01] | 0.86 | [2.20E-03-13.63] |
| p_Firmicutes; c_Clostridia; o_Clostridiales; f_UncClostridiales; g_UncClostridiales | 0.06 | 1.79 | [0.35-4.59] | 0.66 | [3.10E-03-7.17] |
| p_Proteobacteria; c_Betaproteobacteria; o_Burkholderiales; f_Alcaligenaceae; g_Sutterella | 0.86 | 0.72 | [0.3-4.83] | 0.83 | [3.30E-03-3.47] |
| p_Firmicutes; c_Clostridia; o_Clostridiales; f_Lachnospiraceae; g_Lachnospira | 0.72 | 0.7 | [0.14-2.73] | 0.62 | [4.10E-03-6.27] |
| p_Bacteroidetes; c_Bacteroidia; o_Bacteroidales; f_UncBacteroidales; g_UncBacteroidales | 0.01 | 0.63 | [7.40E-04-7.55] | 0 | [0-1.97] |
| p_Firmicutes; c_Clostridia; o_Clostridiales; f_Veillonellaceae; g_Dialister | 0.26 | 0.59 | [0.1-6.76] | 0.6 | [9.30E-04-2.65] |
| p_Cyanobacteria; c_4C0d-2; o_YS2; f_Unc YS2; g_Unc YS2 | 2.40E-03 | 0.53 | [0-5.46] | 0 | [0-1.82] |
| p_Firmicutes; c_Clostridia; o_Clostridiales; f_Lachnospiraceae; g_UncLachnospiraceae | 0.99 | 0.44 | [0.28-1.91] | 0.52 | [0.05-2.85] |
| p_Firmicutes; c_Erysipelotrichi; o_Erysipelotrichales; f_Erysipelotrichaceae; g_Catenibacterium | 0.02 | 0.42 | [4.60E-03-1.95] | 0.1 | [0-8.64] |
| p_Firmicutes; c_Clostridia; o_Clostridiales; f_Lachnospiraceae; g_Roseburia | 0.72 | 0.42 | [0.1-1.66] | 0.4 | [1.90E-03-6.36] |
| p_Firmicutes; c_Clostridia; o_Clostridiales; f_UncClostridiales; g_UncClostridiales | 0.25 | 0.37 | [0.08-1.36] | 0.15 | [4.10E-04-3.7] |
| p_Firmicutes; c_Clostridia; o_Clostridiales; f_Ruminococcaceae; g_Ruminococcus | 0.04 | 0.34 | [0.04-3] | 0.05 | [0-4.8] |
| p_Bacteroidetes; c_Bacteroidia; o_Bacteroidales; f_S24-7; g_Unc S24-7 | 0.13 | 0.28 | [2.30E-03-2] | 0.03 | [0-11.51] |
| p_Bacteroidetes; c_Bacteroidia; o_Bacteroidales; f_Bacteroidaceae; g_Bacteroides | 0.07 | 0.27 | [0.07-14.13] | 1.19 | [0.01-55.27] |
| p_Firmicutes; c_Clostridia; o_Clostridiales; f_Veillonellaceae; g_Mitsuokella | 0.02 | 0.26 | [0-4.34] | 1.30E-03 | [0-3.33] |
| p_Firmicutes; c_Erysipelotrichi; o_Erysipelotrichales; f_Erysipelotrichaceae; g_UncErysipelotrichaceae | 0.04 | 0.23 | [1.40E-03-4.88] | 0.03 | [0-0.61] |
| p_Actinobacteria; c_Actinobacteria; o_Bifidobacteriales; f_Bifidobacteriaceae; g_Bifidobacterium | 0.75 | 0.21 | [0.01-3.94] | 0.23 | [3.60E-03-3.36] |
| p_Firmicutes; c_Clostridia; o_Clostridiales; f_Veillonellaceae; g_Megasphaera | 0.25 | 0.19 | [0-3.4] | 0.01 | [0-2.2] |
| p_Proteobacteria; c_Gammaproteobacteria; o_Enterobacteriales; f_Enterobacteriaceae; g_UncEnterobacteriaceae | 0.41 | 0.18 | [0.02-7.54] | 0.26 | [4.40E-03-27.95] |
| p_Firmicutes; c_Clostridia; o_Clostridiales; f_Lachnospiraceae; g_Coprococcus | 0.17 | 0.17 | [0.03-0.78] | 0.09 | [0-4.79] |
| p_Firmicutes; c_Erysipelotrichi; o_Erysipelotrichales; f_Erysipelotrichaceae; g_Eubacterium | 0.07 | 0.15 | [2.90E-03-0.68] | 0.04 | [0-3.55] |
| p_Firmicutes; c_Clostridia; o_Clostridiales; f_Ruminococcaceae; g_Oscillospira | 0.33 | 0.13 | [0.03-0.75] | 0.13 | [0-5.05] |
| p_Firmicutes; c_Clostridia; o_Clostridiales; f_Lachnospiraceae; g_Blautia | 0.34 | 0.13 | [0.03-0.74] | 0.1 | [0-7.51] |
| p_Firmicutes; c_Clostridia; o_Clostridiales; f_Ruminococcaceae; g_Faecalibacterium | 0.31 | 0.12 | [0.03-0.63] | 0.18 | [6.60E-04-1.22] |
| p_Firmicutes; c_Bacilli; o_Lactobacillales; f_Lactobacillaceae; g_Lactobacillus | 0.25 | 0.11 | [0-1.1] | 0.03 | [1.70E-04-2.84] |
| p_Firmicutes; c_Bacilli; o_Lactobacillales; f_Streptococcaceae; g_Streptococcus | 0.13 | 0.1 | [0.01-0.66] | 0.26 | [0.01-53] |
| p_Firmicutes; c_Clostridia; o_Clostridiales; f_Clostridiaceae; g_Clostridium | 0.98 | 0.09 | [0.01-0.56] | 0.06 | [0-3.16] |
| p_Firmicutes; c_Clostridia; o_Clostridiales; f_Clostridiaceae; g_UncClostridiaceae | 0.41 | 0.08 | [0.01-1.27] | 0.12 | [4.10E-04-1.63] |
| p_Firmicutes; c_Clostridia; o_Clostridiales; f_Lachnospiraceae; g_Ruminococcus | 0.23 | 0.07 | [0.01-0.3] | 0.17 | [2.10E-03-10.18] |
| p_Actinobacteria; c_Coriobacteriia; o_Coriobacteriales; f_Coriobacteriaceae; g_Collinsella | 0.31 | 0.07 | [0.02-0.52] | 0.05 | [2.00E-04-3.68] |
| p_Firmicutes; c_Clostridia; o_Clostridiales; f_Ruminococcaceae; g_UncRuminococcaceae | 0.72 | 0.05 | [4.10E-03-0.31] | 0.01 | [0-0.91] |
| p_Tenericutes; c_Mollicutes; o_RF39; f_Unc RF39; g_Unc RF39 | 1.10E-03 | 0.04 | [2.00E-04-0.98] | 1.70E-04 | [0-0.29] |
| p_Bacteroidetes; c_Bacteroidia; o_Bacteroidales; f_Porphyromonadaceae; g_Parabacteroides | 0.99 | 0.03 | [1.50E-03-0.87] | 0.05 | [0-1.37] |
| p_Actinobacteria; c_Coriobacteriia; o_Coriobacteriales; f_Coriobacteriaceae; g_UncCoriobacteriaceae | 0.03 | 0.03 | [4.00E-03-0.19] | 0.01 | [0-1] |
| p_Bacteroidetes; c_Bacteroidia; o_Bacteroidales; f_Paraprevotellaceae; g_UncParaprevotellaceae | 0.04 | 0.03 | [0-0.36] | 2.00E-04 | [0-0.48] |
| p_Proteobacteria; c_Gammaproteobacteria; o_Pasteurellales; f_Pasteurellaceae; g_Haemophilus | 0.01 | 0.03 | [9.80E-04-0.37] | 0.21 | [4.10E-03-21.85] |
| p_Bacteroidetes; c_Bacteroidia; o_Bacteroidales; f_Rikenellaceae; g_UncRikenellaceae | 0.48 | 0.03 | [1.30E-03-0.8] | 0.02 | [0-1.21] |
| p_Firmicutes; c_Clostridia; o_Clostridiales; f_Lachnospiraceae; g_Dorea | 0.29 | 0.03 | [1.30E-03-0.18] | 0.01 | [0-0.53] |
| p_Firmicutes; c_Clostridia; o_Clostridiales; f_Veillonellaceae; g_Veillonella | 8.10E-05 | 0.01 | [4.90E-04-0.06] | 0.31 | [3.60E-03-23.27] |
| p_Firmicutes; c_Bacilli; o_Turicibacterales; f_Turicibacteraceae; g_Turicibacter | 0.03 | 0.01 | [2.90E-04-1.88] | 8.00E-04 | [0-0.18] |
| p_Firmicutes; c_Clostridia; o_Clostridiales; f_Christensenellaceae; g_UncChristensenellaceae | 0.06 | 0.01 | [0-0.41] | 8.40E-05 | [0-0.16] |
| p_Firmicutes; c_Clostridia; o_Clostridiales; f_Mogibacteriaceae; g_UncMogibacteriaceae | 0.07 | 0.01 | [1.50E-03-0.08] | 3.80E-03 | [0-0.07] |
| p_Proteobacteria; c_Gammaproteobacteria; o_Enterobacteriales; f_Enterobacteriaceae; g_UncEnterobacteriaceae | 0.63 | 0.01 | [4.40E-04-0.27] | 3.30E-03 | [0-1.41] |
| p_Proteobacteria; c_Alphaproteobacteria; o_UncAlphaproteobacteria; f_UncAlphaproteobacteria; g_UncAlphaproteobacteria | 0.02 | 0.01 | [0-7.92] | 6.20E-04 | [0-2.77] |
| p_Lentisphaerae; c_Lentisphaeria; o_Victivallales; f_Victivallaceae; g_UncVictivallaceae | 1.20E-03 | 0.01 | [0-0.2] | 0 | [0-0.02] |
| p_Proteobacteria; c_Deltaproteobacteria; o_Desulfovibrionales; f_Desulfovibrionaceae; g_Bilophila | 0.14 | 0.01 | [2.30E-04-0.1] | 8.70E-04 | [0-0.09] |
| p_Firmicutes; c_Clostridia; o_Clostridiales; f_Lachnospiraceae; g_Anaerostipes | 0.25 | 4.40E-03 | [0-0.03] | 1.30E-03 | [0-0.08] |
| p_Firmicutes; c_Clostridia; o_Clostridiales; f_Veillonellaceae; g_Megamonas | 0.31 | 4.40E-03 | [9.50E-04-1.2] | 0.01 | [0-39.19] |
| p_Actinobacteria; c_Coriobacteriia; o_Coriobacteriales; f_Coriobacteriaceae; g_Slackia | 0.19 | 4.40E-03 | [0-0.06] | 1.50E-03 | [0-0.17] |
| p_Proteobacteria; c_Gammaproteobacteria; o_Pasteurellales; f_Pasteurellaceae; g_UncPasteurellaceae | 0.25 | 4.20E-03 | [0-0.06] | 0.01 | [0-1.66] |
| p_Bacteroidetes; c_Bacteroidia; o_Bacteroidales; f_Odoribacteraceae; g_Odoribacter | 0.30 | 4.00E-03 | [0-0.37] | 1.00E-03 | [0-0.43] |
| p_Firmicutes; c_Clostridia; o_Clostridiales; f_Peptococcaceae; g_UncPeptococcaceae | 0.02 | 3.90E-03 | [0-0.02] | 0 | [0-0.04] |
| p_Proteobacteria; c_Gammaproteobacteria; o_Enterobacteriales; f_Enterobacteriaceae; g_Klebsiella | 0.66 | 2.70E-03 | [2.70E-04-0.05] | 2.00E-03 | [0-1.97] |
| p_Proteobacteria; c_Epsilonproteobacteria; o_Campylobacterales; f_Campylobacteraceae; g_Campylobacter | 0.41 | 1.70E-03 | [0-1.11] | 3.10E-03 | [0-1.84] |
| p_Elusimicrobia; c_Elusimicrobia; o_Elusimicrobiales; f_Elusimicrobiaceae; g_UncElusimicrobiaceae | 0.03 | 1.30E-03 | [0-0.13] | 0 | [0-0.84] |
| p_Actinobacteria; c_Actinobacteria; o_Actinomycetales; f_Micrococcaceae; g_Rothia | 0.06 | 1.30E-03 | [0-3.40E-03] | 3.40E-03 | [0-0.69] |
| p_Bacteroidetes; c_Bacteroidia; o_Bacteroidales; f_Paraprevotellaceae; g_Paraprevotella | 0.05 | 1.30E-03 | [0-0.66] | 0 | [0-0.1] |
| p_Spirochaetes; c_Spirochaetes; o_Spirochaetales; f_Spirochaetaceae; g_Treponema | 0.13 | 1.10E-03 | [0-7.23] | 6.50E-04 | [0-4.29] |
| p_Tenericutes; c_Mollicutes; o_Anaeroplasmatales; f_Anaeroplasmataceae; g_UncAnaeroplasmataceae | 0.08 | 1.10E-03 | [0-0.3] | 4.20E-04 | [0-4.72] |
| p_Firmicutes; c_Clostridia; o_Clostridiales; f_Clostridiaceae; g_UncClostridiaceae | 0.41 | 9.70E-04 | [0-0.02] | 1.60E-03 | [0-0.04] |
| p_Firmicutes; c_Clostridia; o_Clostridiales; f_Veillonellaceae; g_Phascolarctobacterium | 0.91 | 9.50E-04 | [0-0.74] | 1.20E-03 | [0-1.82] |
| p_Proteobacteria; c_Alphaproteobacteria; o_RF32; f_Unc RF32; g_Unc RF32 | 0.02 | 9.30E-04 | [0-1.25] | 0 | [0-7.62] |
| p_Fusobacteria; c_Fusobacteriia; o_Fusobacteriales; f_Fusobacteriaceae; g_Fusobacterium | 0.25 | 8.40E-04 | [0-0.1] | 4.10E-03 | [0-13.74] |
| p_Proteobacteria; c_Gammaproteobacteria; o_Enterobacteriales; f_Enterobacteriaceae; g_Citrobacter | 0.30 | 8.40E-04 | [0-0.38] | 2.00E-04 | [0-0.38] |
| p_Bacteroidetes; c_Bacteroidia; o_Bacteroidales; f_Barnesiellaceae; g_UncBarnesiellaceae | 0.28 | 8.20E-04 | [0-3.55] | 2.70E-04 | [0-1.57] |
| p_Bacteroidetes; c_Bacteroidia; o_Bacteroidales; f_Odoribacteraceae; g_Butyricimonas | 0.04 | 8.20E-04 | [0-0.13] | 0 | [0-0.08] |
| p_Proteobacteria; c_Deltaproteobacteria; o_Desulfovibrionales; f_Desulfovibrionaceae; g_Desulfovibrio | 0.15 | 7.90E-04 | [0-0.08] | 0 | [0-0.3] |
| p_Firmicutes; c_Bacilli; o_Lactobacillales; f_Carnobacteriaceae; g_Granulicatella | 0.05 | 7.10E-04 | [0-0.01] | 2.90E-03 | [0-0.21] |
| p_Proteobacteria; c_Gammaproteobacteria; o_Aeromonadales; f_Succinivibrionaceae; g_UncSuccinivibrionaceae | 5.20E-04 | 6.10E-04 | [0-0.32] | 0 | [0-2.70E-03] |
| p_Tenericutes; c_RF3; o_ML615J-28; f_Unc ML615J-28; g_Unc ML615J-28 | 0.02 | 5.60E-04 | [0-0.71] | 0 | [0-0.26] |
| p_Firmicutes; c_Bacilli; o_Lactobacillales; f_Leuconostocaceae; g_UncLeuconostocaceae | 0.67 | 4.00E-04 | [0-0.05] | 2.10E-04 | [0-0.09] |
| p_Firmicutes; c_Erysipelotrichi; o_Erysipelotrichales; f_Erysipelotrichaceae; g_Holdemania | 0.14 | 3.20E-04 | [0-4.30E-03] | 0 | [0-0.12] |
| p_Firmicutes; c_Erysipelotrichi; o_Erysipelotrichales; f_Erysipelotrichaceae; g_Bulleidia | 0.31 | 2.80E-04 | [0-0.04] | 0 | [0-0.17] |
| p_Firmicutes; c_Bacilli; o_Lactobacillales; f_Enterococcaceae; g_Enterococcus | 0.19 | 2.70E-04 | [0-0.01] | 0 | [0-0.09] |
| p_Firmicutes; c_Clostridia; o_Clostridiales; f_Veillonellaceae; g_Anaerovibrio | 0.24 | 2.60E-04 | [0-1.34] | 0 | [0-4.43] |
| p_Actinobacteria; c_Actinobacteria; o_Actinomycetales; f_Actinomycetaceae; g_Actinomyces | 0.31 | 2.30E-04 | [0-2.30E-03] | 2.90E-04 | [0-0.05] |
| p_Firmicutes; c_Bacilli; o_Lactobacillales; f_Streptococcaceae; g_Lactococcus | 0.33 | 2.00E-04 | [0-0.04] | 0 | [0-0.01] |
| p_Proteobacteria; c_Betaproteobacteria; o_Burkholderiales; f_Oxalobacteraceae; g_Oxalobacter | 0.08 | 2.00E-04 | [0-0.07] | 0 | [0-0.02] |
| p_Proteobacteria; c_Epsilonproteobacteria; o_Campylobacterales; f_Helicobacteraceae; g_Flexispira | 0.01 | 1.90E-04 | [0-1.69] | 0 | [0-0.24] |
| p_Firmicutes; c_Clostridia; o_Clostridiales; f_Veillonellaceae; g_UncVeillonellaceae | 0.03 | 1.00E-04 | [0-0.36] | 0 | [0-2.20E-03] |
| p_Proteobacteria; c_Betaproteobacteria; o_Neisseriales; f_Neisseriaceae; g_Neisseria | 0.11 | 5.00E-05 | [0-0.01] | 8.10E-04 | [0-0.57] |
| p_Proteobacteria; c_Gammaproteobacteria; o_Enterobacteriales; f_Enterobacteriaceae; g_Erwinia | 0.96 | 4.50E-05 | [0-2.10E-03] | 0 | [0-0.1] |
| p_Proteobacteria; c_Gammaproteobacteria; o_Pasteurellales; f_Pasteurellaceae; g_Actinobacillus | 0.04 | 0 | [0-3.60E-03] | 9.00E-04 | [0-1.77] |
| p_Proteobacteria; c_Gammaproteobacteria; o_Pasteurellales; f_Pasteurellaceae; g_Aggregatibacter | 0.25 | 0 | [0-1.30E-03] | 2.00E-04 | [0-0.4] |
| p_Verrucomicrobia; c_Verrucomicrobiae; o_Verrucomicrobiales; f_Verrucomicrobiaceae; g_Akkermansia | 0.64 | 0 | [0-0.04] | 0 | [0-2.59] |
| p_Proteobacteria; c_Gammaproteobacteria; o_Aeromonadales; f_Succinivibrionaceae; g_Anaerobiospirillum | 0.71 | 0 | [0-3.80E-03] | 0 | [0-1.49] |
| p_Spirochaetes; c_Brachyspirae; o_Brachyspirales; f_Brachyspiraceae; g_Brachyspira | 0.26 | 0 | [0-0.39] | 0 | [0-0.15] |
| p_Proteobacteria; c_Betaproteobacteria; o_Burkholderiales; f_Comamonadaceae; g_Comamonas | 0.29 | 0 | [0-0.08] | 0 | [0-0.12] |
| p_Proteobacteria; c_Gammaproteobacteria; o_Enterobacteriales; f_Enterobacteriaceae; g_Morganella | 0.11 | 0 | [0-0] | 0 | [0-0.32] |
| p_Firmicutes; c_Erysipelotrichi; o_Erysipelotrichales; f_Erysipelotrichaceae; g_p-75-a5 | 0.07 | 0 | [0-1.3] | 0 | [0-0.01] |
| p_Proteobacteria; c_Betaproteobacteria; o_Burkholderiales; f_Burkholderiaceae; g_Pandoraea | 0.03 | 0 | [0-4.90E-03] | 0 | [0-0.06] |
| p_Firmicutes; c_Erysipelotrichi; o_Erysipelotrichales; f_Erysipelotrichaceae; g_RFN20 | 0.05 | 0 | [0-0.64] | 0 | [0-0.02] |
| p_Bacteroidetes; c_Bacteroidia; o_Bacteroidales;f_UncBacteroidales; g_UncBacteroidales | 0.91 | 0 | [0-0.19] | 0 | [0-0.12] |
| p_Verrucomicrobia; c_Opitutae; o_Cerasicoccales; f_Cerasicoccaceae; g_UncCerasicoccaceae | 0.07 | 0 | [0-0.12] | 0 | [0-0.02] |
| p_Proteobacteria; c_Gammaproteobacteria;o_UncGammaproteobacteria;f_UncGammaproteobacteria; g_UncGammaproteobacteria | 0.10 | 0 | [0-0.79] | 2.00E-04 | [0-0.51] |
| p_Firmicutes; c_Bacilli; o_Gemellales; f_Gemellaceae; g_UncGemellaceae | 0.14 | 0 | [0-1.90E-03] | 1.70E-04 | [0-0.04] |
| p_Firmicutes; c_Clostridia; o_Clostridiales; f_Veillonellaceae; g_UncVeillonellaceae | 0.14 | 0 | [0-0.42] | 0 | [0-0] |

P values are shown as Benjamini-Hochberg’s false discovery rate (FDR)
